# Lymphocyte access to lymphoma is impaired by high endothelial venule regression

**DOI:** 10.1016/j.celrep.2021.109878

**Published:** 2021-10-26

**Authors:** Lutz Menzel, Maria Zschummel, Tadhg Crowley, Vedran Franke, Michael Grau, Carolin Ulbricht, Anja Hauser, Volker Siffrin, Marc Bajénoff, Sophie E. Acton, Altuna Akalin, Georg Lenz, Gerald Willimsky, Uta E. Höpken, Armin Rehm

**Affiliations:** 1 Translational Tumorimmunology, Max-Delbrück-Center for Molecular Medicine Berlin, Germany, 13125 Berlin, Germany; 2 Microenvironmental Regulation in Autoimmunity and Cancer, Max-Delbrück-Center for Molecular Medicine Berlin, 13125 Berlin, Germany; 3 Neuroimmunology Laboratory, Max-Delbrück-Center for Molecular Medicine Berlin, Germany, 13125 Berlin, Germany; 4 Bioinformatics & Omics Data Science Platform, BIMSB at Max-Delbrück-Center for Molecular Medicine Berlin, 13125 Berlin, Germany; 5 Medical Department A for Hematology, Oncology, and Pneumology, University Hospital Münster, 48149 Münster, Germany; 6 Charité-Universitätsmedizin Berlin, corporate member of Freie Universität Berlin and Humboldt-Universität zu Berlin, Department of Rheumatology and Clinical Immunology, and Immune Dynamics, Deutsches Rheumaforschungszentrum Berlin, 10117 Berlin, Germany; 7 Neuroimmunology Laboratory, Experimental and Clinical Research Center, Charité-Universitätsmedizin Berlin, 13125 Berlin, Germany; 8 Aix Marseille University, CNRS, INSERM, Centre d’Immunologie de Marseille-Luminy, 13288 Marseille, France; 9 Stromal Immunology Group, MRC Laboratory for Molecular Cell Biology, University College London, WC1E 6BT London, UK; 10 Institute of Immunology, Charité-Universitätsmedizin Berlin, corporate member of Freie Universität Berlin and Humboldt-Universität zu Berlin, 13125 Berlin, Germany; 11 German Cancer Research Center, 69120 Heidelberg, Germany; 12 German Cancer Consortium, partner site Berlin, Germany

**Keywords:** B cell lymphoma, high endothelial venules, blood endothelial cells, immunosurveillance, angiogenesis, tumor stroma, immune cell trafficking, chemokines, lymphotoxin beta-receptor, dendritic cells

## Abstract

Blood endothelial cells display remarkable plasticity depending on the demands of a malignant microenvironment. While studies in solid tumors focus on their role in metabolic adaptations, formation of high endothelial venules (HEVs) in lymph nodes extends their role to the organization of immune cell interactions. As a response to lymphoma growth, blood vessel density increases; however, the fate of HEVs remains elusive. Here, we report that lymphoma causes severe HEV regression in mouse models that phenocopies aggressive human B cell lymphomas. HEV dedifferentiation occurrs as a consequence of a disrupted lymph-carrying conduit system. Mechanosensitive fibroblastic reticular cells then deregulate CCL21 migration paths, followed by deterioration of dendritic cell proximity to HEVs. Loss of this crosstalk deprives HEVs of lymphotoxin-β-receptor (LTβR) signaling, which is indispensable for their differentiation and lymphocyte transmigration. Collectively, this study reveals a remodeling cascade of the lymph node microenvironment that is detrimental for immune cell trafficking in lymphoma.

## Highlights

• Aggressive B-cell-lymphoma-induced immunosuppressive niche in lymph nodes
• Lymphoma causes a vascular remodeling cascade within the lymph node stroma
• Loss of DC-HEV crosstalk deprives blood endothelial cells of LTβR signaling
• Lymphoma-induced HEV dedifferentiation is detrimental for immune cell trafficking

## Graphical abstract

**Figure:**
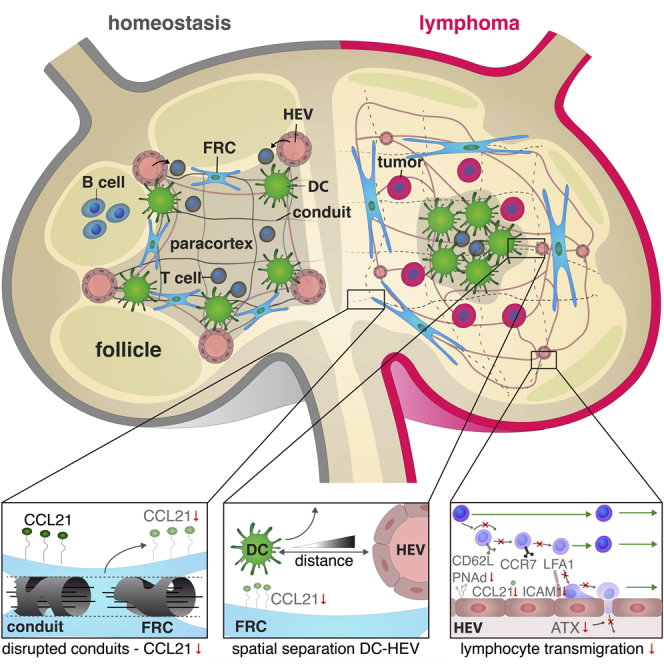

## Teaser

Menzel et al. report high endothelial venules (HEVs) regression that is detrimental for immune cell trafficking in lymph nodes with lymphoma. HEV dedifferentiation occurs as a consequence of a disrupted conduit system and impairment of chemokine-guided lymphocyte migration. Loss of dendritic cell-HEV interaction abrogates lymphotoxin β-receptor signaling, required for HEV maintenance.

## Introduction

Lymph nodes (LNs) integrate two vascular systems to maintain homeostasis, comprising blood and lymphatic vessels (Drayton et al., 2006; Liao and Ruddle, 2006). Not only do blood vessels provide the local environment with import of nutrients and removal of metabolites, but vessel-forming blood endothelial cells (BECs) also differentiate into high endothelial venules (HEVs) and endow LNs with immunocompetence. Only if lymphocyte import is synchronized with lymphatic vessel-dependent transport of antigen and antigen-presenting cells can a spatial proximity of these cell types ensure proper priming of immune cells (Bajénoff et al., 2003).

HEVs are postcapillary venules, which furnish lymphocyte transmigration routes. To fulfill their pivotal role in lymphocyte homing, mature HEVs must be equipped with peripheral node addressin (PNAd), which engages with lymphocyte-expressed L-selectin, followed by a process referred to as tethering and rolling (Girard et al., 2012). The homeostatic chemokine CCL21 expressed and immobilized on HEVs recruits naive and central memory T cells via the CCR7 receptor. This leads to integrin αLβ2 (LFA-1) affinity enhancement, lymphocyte arrest, and transendothelial migration (Girard et al., 2012). The importance of this dynamic system has been elucidated under homeostatic conditions, and further insights into the remodeling and molecular factors involved were obtained from infection models (Guarda et al., 2007; Veerman et al., 2019).

HEVs show a high degree of plasticity, as they undergo rapid phenotypical changes in response to microenvironmental stimuli, including growth, regression, and restoration during inflammation (Liao and Ruddle, 2006; Kumar et al., 2010; Mondor et al., 2016). Lymphotoxin-β-receptor (LTβR) signaling via the non-canonical nuclear factor κB (NFκB) pathway is a prerequisite for the formation and maintenance of HEVs (Drayton et al., 2004; Onder et al., 2013). Mature HEVs rapidly dedifferentiate during pharmacological inhibition of the LTβR (Browning et al., 2005; Onder et al., 2013) or as a consequence of *in vivo* dendritic cell (DC) depletion, which provides the ligands lymphotoxin-α_1_β_2_ (LTα_1_β_2_) and LIGHT (lymphotoxin-like, exhibits inducible expression, and competes with herpes simplex virus glycoprotein D (gD) for HVEM, a receptor expressed by T lymphocytes) (Moussion and Girard, 2011). As a hint to their mechanosensitive properties, the integrity of HEVs is strongly compromised during solid stress in metastatic LNs (Jones et al., 2021) and in LNs after surgical or pharmacological deprivation of the afferent lymph flow (Hendriks and Eestermans, 1983; Mebius et al., 1991; Chang et al., 2019). Lymph flow is carried from the lymphatic sinus through the LN parenchyma and transferred to the HEV lumen by a reticular conduit system embedded within the fibroblastic reticular cell (FRC) network (Gretz et al., 2000; Reynoso et al., 2019). FRCs serve as a scaffold in this network, but they are also sensitive to changes of the lymph flow, responding with transcriptional deregulation of homeostatic chemokines and breakdown of the HEV support (Tomei et al., 2009; Chang et al., 2019).

Despite their origin as autochthonous LN tumors, very little is known about the fate of HEVs in the context of B cell lymphoma (BCL). An important blood vessel pathology is the angiogenic increase of the microvessel density (MVD) (Cardesa-Salzmann et al., 2011; Gloger et al., 2020; Menzel et al., 2020), which is prognostically unfavorable in diffuse large BCL (DLBCL). A previous immunohistology study found reduced numbers of HEVs with a damaged appearance in high-grade B cell non-Hodgkin lymphoma (B-NHL), while in low-grade tumors, HEVs were preserved (Pajor et al., 1990). Considering that emerging immunotherapies are dependent on adequate trafficking routes for naive or engineered T cells to combat lymphoma (Ansell and Lin, 2020), we sought to interrogate the molecular and cellular factors that contribute to the loss of functional HEV structures. Here, we report a severe loss of HEVs in two independent aggressive lymphoma mouse models, a phenotype that we validated in human DLBCL. While it seems clear that mouse models can not exactly phenocopy B cell origin, phenotype, and genetics of defined human lymphoma entities, they are suitable to mimic features like growth kinetics, homing, migration, and dependency on a non-malignant LN infrastructure (O’Connor et al., 2019; Menzel et al., 2020). Furthermore, they provide insight into processes at tumor onset in an *in vivo* setting, whereas human specimens are usually obtained from established and progressed lymphoma with a severely disturbed LN microanatomy.

In the lymphoma-induced LN remodeling cascade, HEV dedifferentiation and loss of functionality emerged as a consequence of disturbed conduit channeled lymph flow, aberrant extracellular matrix (ECM) deposition, and the subsequent deregulation of CCL21 intranodal DC migration routes. Functionally impaired HEVs failed to support efficient T lymphocyte endothelial transmigration and, thus, compromise the capacity to support effective immune cell cooperation. We not only provide mechanistical insights into a lymphoma-related loss of HEV integrity, but also address therapeutic solutions for improved immunotherapies.

## Results

### Lymphoma-induced LN expansion relies on differential induction of BEC subsets

**Figure 1:**
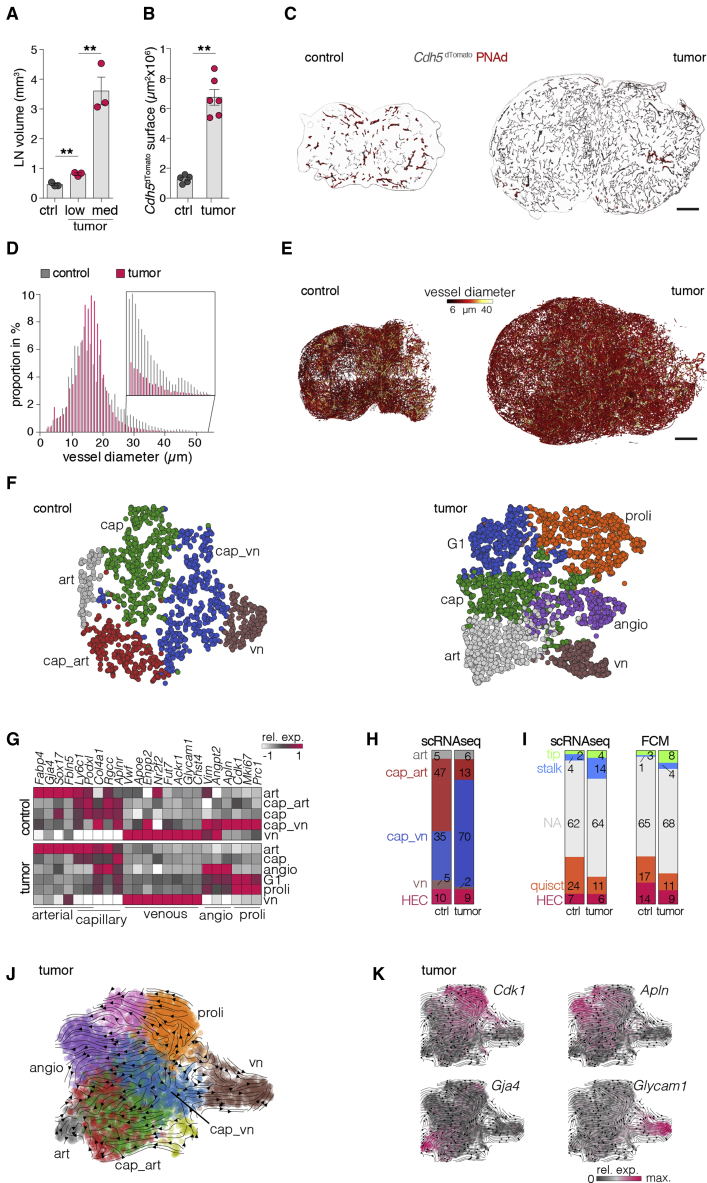
Lymphoma induces LN expansion and increased density of capillary-like blood vessels (A) Quantification of LN volume using light sheet microscopy (LSM). n = 3 mice per group. (B) Blood vessel surface quantification in sections of *Cdh5*^dTomato^ reporter mice. n = 5–6 mice per group. (C) Representative segmentation of LN sections of *Cdh5*^dTomato^ reporter mice stained for PNAd. (D) Diameter distribution of vessel segments in LNs from *Cdh5*^dTomato^ reporter mice. (E) 3D reconstruction of the whole-LN vascular tree, recorded by LSM. Color scale depicts mean diameter of segments. (F) t-distributed stochastic neighbor embedding (t-SNE) dimensional reduction plots of control (n = 1,545) and medium tumor (n = 3,915) BECs analyzed by scRNA-seq and segregated by unsupervised clustering in arterial (art), venoular (vn), capillary (cap), angiogenic (angio), G1 phase (G1), and proliferation (proli) clusters. (G) Expression of BEC subset signature genes displayed as mean gene expression per subset. (H) Proportions of BEC subsets from unsupervised clusters; numbers indicate the percentages of subsets among all BECs. (I) Frequencies in percent of BECs annotated to subsets along known markers: (left) scRNA-seq datasets and (right) analyzed by flow cytometry (FCM). (J and K) UMAP dimensional reduction plots of BECs from tumor LNs depicted in unsupervised clusters (J) or depicting indicated gene expression (K). Vectors represent predicted trajectories based on RNA-velocity computation. Expression intensities indicated by color scale (G and K). Scale bars, 100 μm (C and E). Mean and SEM are indicated. Significances calculated by t test (A) or Mann-Whitney U-test (B). ^∗∗^p < 0.01.

The mechanisms and kinetics of inflammation-induced LN expansion are well described, whereas lymphoma-associated LN expansion correlating with tumor progression remains to be elucidated. We modeled aggressive human BCL using transplantable Eμ-*Myc* murine lymphoma cells (Reimann et al., 2010; Rehm et al., 2011). Control cells were obtained from tumor-naive mice. We defined an early-stage tumor (low tumor, 5%–15% Eμ-*Myc* of CD45^+^ cells) and a progressed tumor stage (30%–50% Eμ-*Myc* cells, referred to as medium tumor or solely “tumor”) (Figures 1A, S1A, and S1B). Assessment of the vasculature revealed a multi-fold expansion with an overrepresentation of capillary-like vessels (Figures 1B and 1C). Larger vessels occurred in lower frequencies in tumor LNs (Gloger et al., 2020) (Figures 1D and 1E).

To elaborate cellular subsets of vessel-forming endothelia, we performed single-cell mRNA sequencing (scRNA-seq) analysis of isolated BECs (Figure S1C), covering 1,545 (control) and 3,915 cells from LNs with medium tumor. Unsupervised clustering defined five BEC subsets with distinct gene expression in control LNs, including three capillary (cap, cap_vn, cap_art), venous (vn), and arterial (art) subsets; in tumor-challenged LNs, six subsets were defined as proliferative (prol), art, cap, vn, cells in G1 phase (G1), and angiogenic (angio) (Figures 1F, 1G, and S1D). For comparison of both groups, single-cell affiliations were identified with respect to known subset markers (arterial [art]: *Sox17*, *Gja4*, *Rbp7*; arterial capillaries [cap_art]: *Rgcc*, *Ly6c1*, *Ramp3*; venous capillaries [cap_vn]: *Enpp2*, *Col4a1*, *Aplnr*; venous [vn]: *Lrg1*, *Vwf*, *Il6st*, *Chst4*^neg^; HEV: *Glycam1*, *Chst4*) (Brulois et al., 2020; Kalucka et al., 2020) (Figure S1E). The proportion of cap_vn was strongly increased in tumor-challenged LNs, while BECs from controls included a similar frequency of cap_art and cap_vn (Figure 1H). We further differentiated cell clusters applying key genes associated with angiogenesis-related subsets (Zhao et al., 2018) (tip [tip]: *Esm1*, *Cxcr4*; stalk [stalk]: *Jag1*, *Hey1*; quiescent [quiescent]: *Cd36*, *Flt1*, *Mki67*^*neg*^, *Cdk1*^*neg*^; and HEV cells [HECs]: *Glycam1*, *Chst4*). Very few tip and stalk cells were found in control LNs, while the majority of cells remained quiescent or could not be conclusively attributed (NA). Tumors induced tip and stalk cell differentiation and a decrease of endothelial quiescence (Figure 1I). Flow cytometry (FCM) analysis confirmed BEC subset proportions along surface marker expression of tip (CXCR4), stalk (JAG-1), HEV (PNAd) and quiescent (CD36) cells (Figures 1I, S1C, and S1F). Trajectories based on RNA velocity analysis revealed that cap_vn cells constituted a proliferative progenitor subset, with potential to differentiate into the aforementioned subsets (Figures 1J and 1K). Furthermore, a fluorescent fate-mapping mouse model (*Cdh5*^Ubow^) conditionally labels mature ECs with different fluorophores in a stochastic manner. Equally dividing endothelial cells (ECs) during LN angiogenesis appear in small multi-colored segments, whereas proliferation of a few highly proliferative cells generates large monocolored vessels (Mondor et al., 2016). We found larger clonally related segments of vasculature composed of monocolored cells within tumor HEVs, whereas classical BEC (cBEC) clusters were only doubled in size compared to controls (Figure S1G). Taken together, these results indicate a strong proliferative capacity of rather undifferentiated capillary venous ECs, whereas few cells in HEVs act as local progenitors during expansion.

### Lymphoma progression in LNs causes severe regression of HEVs

HEV-forming HECs responded to lymphoma-induced angiogenic and proliferative stimuli; however, vessels with larger diameters, a phenotypical feature of HEVs, were decreased.

**Figure 2:**
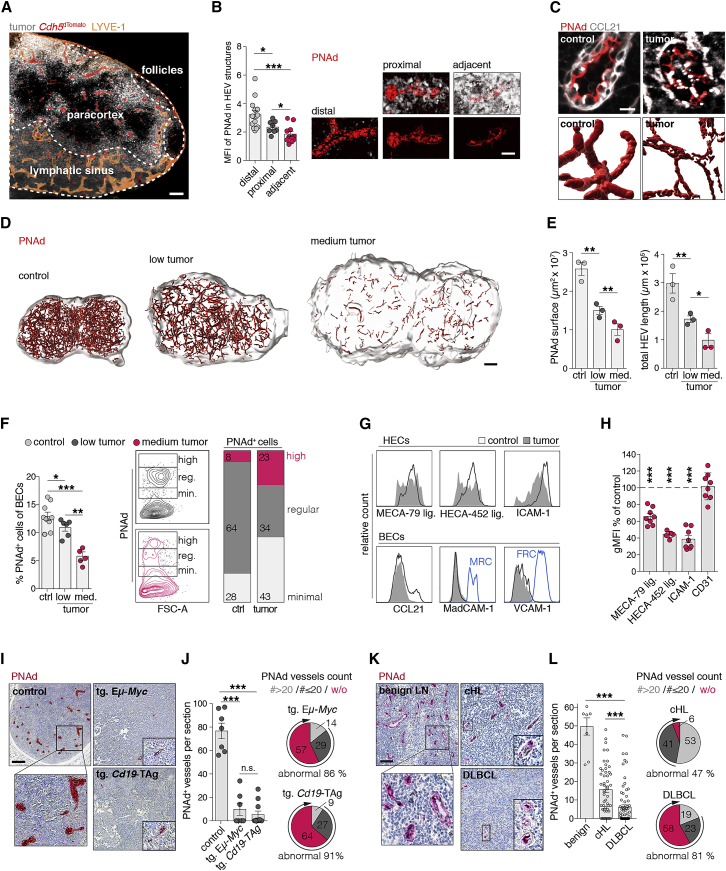
Lymphoma growth causes progressive regression of HEVs (A) Representative LN section from *Cdh5*^dTomato^ mice with Eμ-*Myc* tumor cells^CFP^ (low tumor) and LYVE-1 staining. (B) PNAd expression relative to tumor cell distance; mean fluorescence intensity (MFI), n = 3 mice per group. (C) PNAd expression in HEVs (top) and PNAd^+^ surface rendering in LNs with medium tumor load (bottom). (D) Whole-LN PNAd^+^ expression, visualized by i.v. administered MECA-79 antibody, recorded by LSM. (E) Quantification of PNAd^+^ surface area and vessel length in LSM datasets. n = 3 mice per group. (F) FCM quantification of PNAd^+^ HECs relative to all BECs. Expression levels grouped as minimal, min.; regular, reg.; and high. Ctrl n = 9, low n = 6, and medium tumor n = 5 mice. (G and H) Histograms of HEC markers in FCM analysis (G) and gMFI values as percentage relative to controls (H). n = 6–8 mice per group. As positive controls, MRCs and FRCs (blue line) are included. (I) Immunhistochemical detection of PNAd expression in LNs from control and diseased transgenic (tg.) Eμ-*Myc* or tg. *Cd19*-TAg mice. (J) Left: count per whole LN section of PNAd^+^ structures. Right: pie charts indicate cases grouped by count of PNAd^+^ vessels per section (# > 20, # ≤ 20, w/o). Sections w/o or PNAd^+^ vessel count <20 were considered “abnormal.” Controls n = 5, tg. Eμ-*Myc* n = 11, tg. *Cd19*-TAg n = 9 mice per group. (K) Immunhistochemical detection of PNAd expression in benign human LNs or with DLBCL or cHL. (L) PNA^+^ vessels per section, as in (J). Benign LNs n = 7, cHL n = 51, DLBCL n = 79. Scale bars, 100 μm (A, D, I, and K), 20 μm (B), and 10 μm (C). Mean and SEM are indicated. Data points represent individual HEVs (B), mice (D, F, H, and J) or cases (L). Statistics were calculated using Mann-Whitney U-test (B, F, J, and L), Wilcoxon rank sum-test (H), and t test (D). ^∗^p < 0.05, ^∗∗^p < 0.01, ^∗∗∗^p < 0.005.

Consistent with a leading function of CCR7 in lymphoma cell homing (Rehm et al., 2011), Eμ-*Myc* cells were preferentially located within proximity to HEVs, at the border of the paracortex and in the cortical ridge (Figures 2A and S2A–S2C). We observed a gradual decrease of PNAd expression depending on the proximity of vessels to tumor cells (Figure 2B). In controls, PNAd expression was evenly distributed at the luminal side of HEVs, whereas tumor (medium tumor)-challenged LNs exhibited a heterogenous distribution (Figure 2C). Whole-LN assessment of HEVs, labeled by intravenously (i.v.) infused anti-PNAd (MECA-79) antibodies, revealed a severely reduced and incoherent HEV network (Figure 2D). The intraluminal PNAd surface coverage and overall HEV network length was reduced (Figure 2E). The proportion of high endothelial cells (HECs) (PNAd^+^ BECs) decreased depending on the disease stage, with a more severe reduction in advanced tumors. Although in lymphoma a few BECs still exhibited high PNAd expression, the vast majority expressed low or no PNAd on their surfaces; therefore, HECs lost an essential feature of their phenotype, resulting in a greater heterogeneity of the remaining HECs (Figure 2F). In support of HEV dedifferentiation, we found not only a decrease of PNAd antigen defined by MECA-79 and HECA-452 recognition, but also a downregulation of ICAM-1 and CCL21. MadCAM-1 and VCAM-1 were not detected in BECs (Figures 2G and 2H). Consistent with the lymphoma transplantation model, a severe loss (57%) or abnormally low numbers (29%, count ≤ 20 per section) of HEVs was apparent in diseased transgenic Eμ-*Myc* mice (tg. Eμ-*Myc*; [abnormal HEV count in 86% of cases, 6/7]). Likewise, transgenic mice that spontaneously develop BCLs driven by the SV40 large T oncogene (referred to as tg. *Cd19*-TAg) (Hoser et al., 2018) developed a similar HEV phenotype (abnormal HEV count in 91% of cases, 10/11), suggesting that vascular alterations were not caused by *Myc* oncogene activity. Notably, spontaneously developed tumors were typically more advanced compared to the transfer model (>80% tumor cells) (Figures 2I and 2J). To investigate whether human lymphomas phenocopy these HEV alterations, we chose two different aggressive lymphoma entities—DLBCL and Burkitt’s lymphoma(BL)—and, additionally, classical Hodgkin’s lymphoma (cHL), which is a striking example of the predominance of benign immune cells and a regulatory role of the stroma. A total loss or abnormally low numbers of PNAd^+^ structures were observed in 81% of DLBCL specimens (64/79) and in both BL cases investigated. Contrarily, HEVs were much better preserved in cHL (w/o or low count 47%; 24/51) (Figures 2K, 2L, and S2D).

Taken together, loss of phenotypic HEV markers in the murine lymphoma models mimics essential features of vascular alterations in aggressive human B-NHLs.

### Gradual loss of HEC-specific gene expression pattern in lymphoma

HEVs are composed of heterogeneous HECs (Veerman et al., 2019), and the state of HEC maturity and differentiation is critically dependent on the continuous interaction with their microenvironment (Lacorre et al., 2004; Moussion and Girard, 2011). We used scRNA-seq of BECs to resolve the processes that led to the loss of HEVs during high-grade lymphoma.

**Figure 3:**
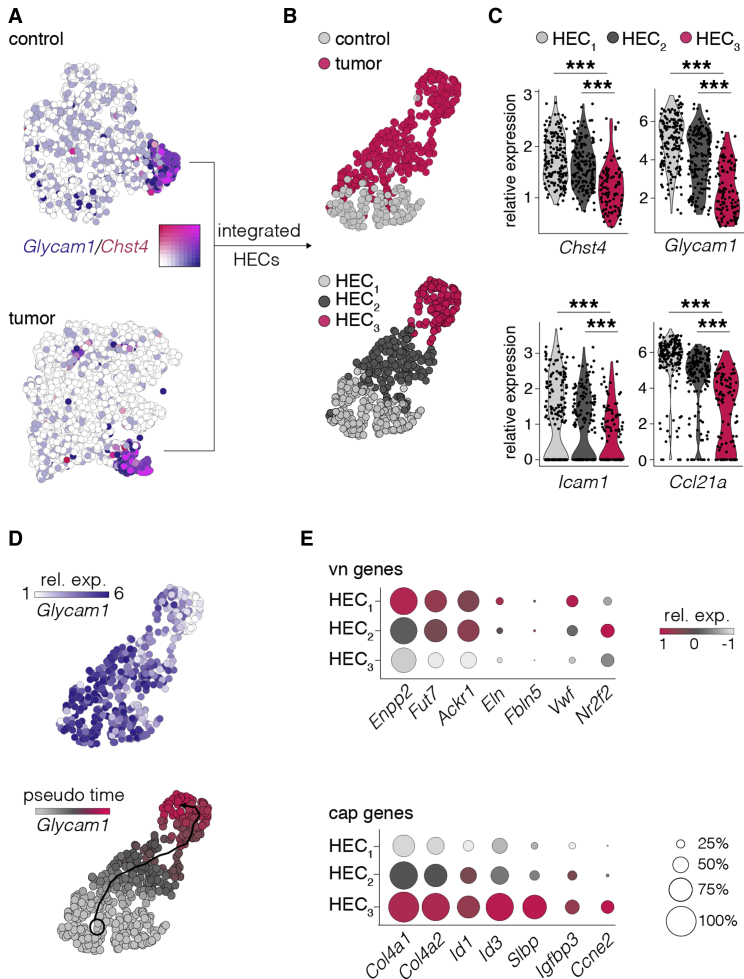
Gradual loss of the phenotypic HEV transcriptome program during tumor growth (A) t-SNE plots depicting gene expression of HEV-specific genes, illustrating integration of control and tumor scRNA-seq datasets and selection of HECs (*Glycam*^+^ and *Chst4*^+^) for further analysis. (B) Uniform manifold approximation and projection (UMAP) dimensional reduction of (top) HECs (color depicts affiliation to the condition group) and (bottom) populations from unsupervised clustering. (C) Violin plots of lymphocyte transmigration-associated gene expression in the HEC subpopulations. (D) UMAP dimensional reduction of (top) HECs (color scaled for relative gene expression) or (bottom) pseudotime expression of *Glycam1*. (E) Dot plots depict selected genes indicative of venous (vn) or capillary (cap) identity. Expression differences are depicted according to color scale, and percentage of expressing cells is represented by the dot diameter. Significance calculated with Wilcoxon rank sum test (C). ^∗∗^p < 0.005.

The vn clusters included HECs and non-HEC venous cBECs. HECs were selected according to *Glycam1* and *Chst4* expression (Lee et al., 2014) in the integrated dataset of both conditions (Figures 3A, S3A, and S3B). Dimensional reduction demonstrated a minor overlap of HECs from both groups (Figure 3B, top), with several genes differentially expressed (Figure S3C). Using unsupervised clustering to account for the heterogeneity of HEC transcriptomes independent of the conditional affiliation, three HEC clusters (HEC_1-3_) were identified (Figures 3B, bottom, and S3D). The HEC_1_ subset included most of the cells from control LNs, whereas the majority of HEC_2_ and HEC_3_ were derived from tumor LNs (Figure S3E). In keeping with the phenotypic loss of HEVs in tumor LNs, the expression levels of *Glycam1*, *Chst4*, *Icam1*, and *Ccl21a* were progressively reduced in HEC_1_ to HEC_3_ (Figure 3C), similar to the reduction of gene expression of PNAd producing glycan-generating enzymes (Lee et al., 2014) (Figures S3F and S3G). Genes associated with an activated inflammatory endothelium (Guarda et al., 2007; Veerman et al., 2019) like *CXCL9*, *Vcam1*, *Sele* (CD62E), and *Selp* (CD62P) were not detected or were weakly expressed (Figure S3H).

Pseudotime trajectory analysis along *Glycam1* expression depicted HEC_1_ as the starting point of the dedifferentiation (Figure 3D). In support of HEC_2_ as an intermediate differentiation state, a ternary plot, which depicts cluster-associated restriction or enrichment of genes, showed that HEC_1_ and HEC_3_ had only a few genes in common, while HEC_2_ filled a transition state (Figure S3I). Gene set enrichment analysis revealed differentially regulated pathways associated with transendothelial migration, cell adhesion, and NFκB signaling as enriched in HEC_1_. HEC_3_ was characterized by enrichment of gene sets associated with a loss of cellular quiescence, Burkitt’s lymphoma, and DLBCL (Figure S3I). The differential expression of several genes signifying venous or capillary cell affiliation suggested a dedifferentiation of prior venous HECs toward an immature capillary-like phenotype (Figure 3E). Alternatively, newly attracted precursor ECs with a low-grade HEC differentiation might account for the loss of phenotypic HEV structures. Collectively, differential gene representation in HEC clusters suggested that HEVs exposed to aggressive lymphoma exhibited a remarkable plasticity, which resulted in a gradual loss of phenotypic HEC gene expression.

### Lymphoma induces a non-permissive HEV condition for lymphocyte immigration

**Figure 4:**
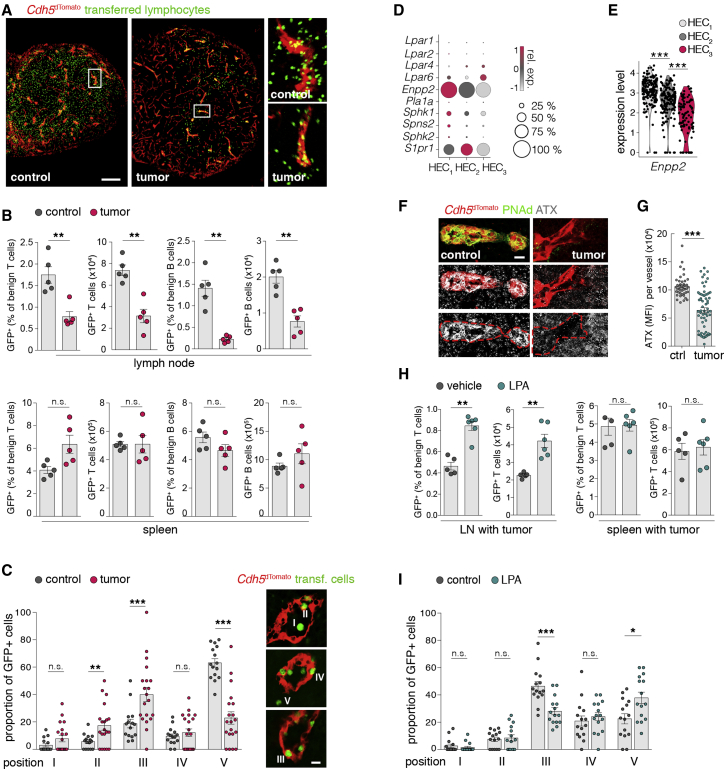
HEVs are functionally impaired in supporting lymphocyte transmigration (A) LN sections from *Cdh5*^dTomato^ reporter mice after adoptive transfer of lymphocytes. Boxes indicate magnified areas. (B) Quantification of LN and spleen infiltrating lymphocytes by FCM. Gating strategy is given in Figure S4A. N = 5 mice per group. (C) Transmigration phase of transferred lymphocytes. Positions at HEVs were defined as (1) lumen, (2) attached to surface, (3) pockets, (4) at basal lamina, and (5) in parenchyma. N = 5 mice per group. Bottom: representative images depicting positions. (D) Dot plot representing the expression of genes associated with lymphocyte immigration from HEV pockets over the basal lamina. (E) Violin plots depicting *Enpp2* (ATX) gene expression in single cells of HEC clusters. (F) LN sections from *Cdh5*^dTomato^ reporter mice stained for autotaxin (ATX) and PNAd. (G) MFI of ATX staining in *Cdh5*^dTomato^ vessels. n = 3 mice per group. (H) FCM analysis of LN and spleen infiltrating T cells. Before T cell transfer, cells were pretreated with lysophosphatidic acid (LPA) or vehicle. N = 5–6 mice per group. (I) Microscopic analysis was applied to assess the transmigration phase of transferred lymphocytes supplemented with LPA or vehicle (as in C). N = 3 mice per group. Scale bars, 100 μm (A) and 10 μm (C and F). Mean and SEM are indicated. Data points represent individual mice (B, H, and K) or vessels (C, G, and I). Statistics were calculated with Mann-Whitney U-test (B, C, and G–I) or Wilcoxon rank sum test (E). ^∗^p < 0.05; ^∗∗^p < 0.01; ^∗∗∗^p < 0.005; n.s., not significant.

Lymphoma-induced effects on lymphocyte recirculation are elusive. However, a prerequisite for immunosurveillance is an intact system of lymphocyte attraction and LN immigration. To determine the functional consequences of the reduced functional HEV markers, we adoptively transferred lymphocytes (GFP^+^) into control mice and recipients with tumor. Homing of T and B lymphocytes to peripheral LN parenchyma with tumor was severely impaired, whereas immigration to the spleen remained unchanged, indicating an HEV-associated transmigration defect (Figures 4A, 4B, and S4A). Activated T cells transiently downregulated the expression of the LN homing receptors CCR7 and CD62L and are therefore excluded from LN infiltration via the HEV route during physiological conditions (Figure S4B). Reactive HEVs express inflammation-related receptors and chemokines (e.g., CXCL9, CD62E/*Sele*, CD62P/*Selp*) (Guarda et al., 2007; Veerman et al., 2019). Here, the exclusion of transplanted activated T cells from LNs in mice with tumor supported our findings that blood vessels during lymphoma are different from those of reactive LNs (Figure S4C). To dissect the transmigration route of lymphocytes accessing the HEV environment, we analyzed their numbers and localization. Positions were defined as (1) HEV lumen, (2) attached to luminal side, (3) HEV pockets, (4) abluminal side, and (5) parenchyma (Wendland et al., 2011). Adoptively transferred lymphocytes in tumor-bearing mice were predominantly restrained to HEV pockets, whereas in controls, the vast majority of lymphocytes entered the parenchyma (Figure 4C).

Extended lymphocyte residency within HEV pockets correlated with a reduced expression of *Enpp2* in HEC_3_, a gene encoding for the ectoenzyme autotaxin (ATX) (Figure 4D). ATX produces the lipid second messenger lysophosphatidic acid (LPA) from lysophosphatidylcholine (LPC) and is crucial for the constitutive lymphocyte migration from pockets across the basal lamina of HEVs (Kanda et al., 2008; Bai et al., 2013). HEVs in control LNs consistently expressed ATX, in contrast to a reduced and highly variable expression in tumors and HEC subsets, respectively (Figures 4E–4G and S4D). To validate the functional role of ATX, treatment of lymphocytes with LPA during cell transfer partly compensated the lymphoma-induced homing impairment of T cells (Figure 4H). In line, LPA treatment substantially reduced lymphocyte retention within HEV pockets (Figure 4I). Adoptively transferred naive CD4^+^ OT-II T cells (CD44^low^) showed T cell receptor (TCR)-specific activation in control and tumor LNs after immunization with ovalbumin; however, the strongly impaired T cell homing to LNs with tumor was also associated with a decreased number of activated CD4^+^OT-II^+^CD44^high^ cells (Figure S4E and S4F).

Taken together, the molecular definition of HEV dedifferentiation corresponded to a functional loss of competence, since tumor-exposed LNs failed to support efficient lymphocyte transmigration.

### Tumor-associated spatial separation between DCs and HEVs prevents LTβR signaling

**Figure 5:**
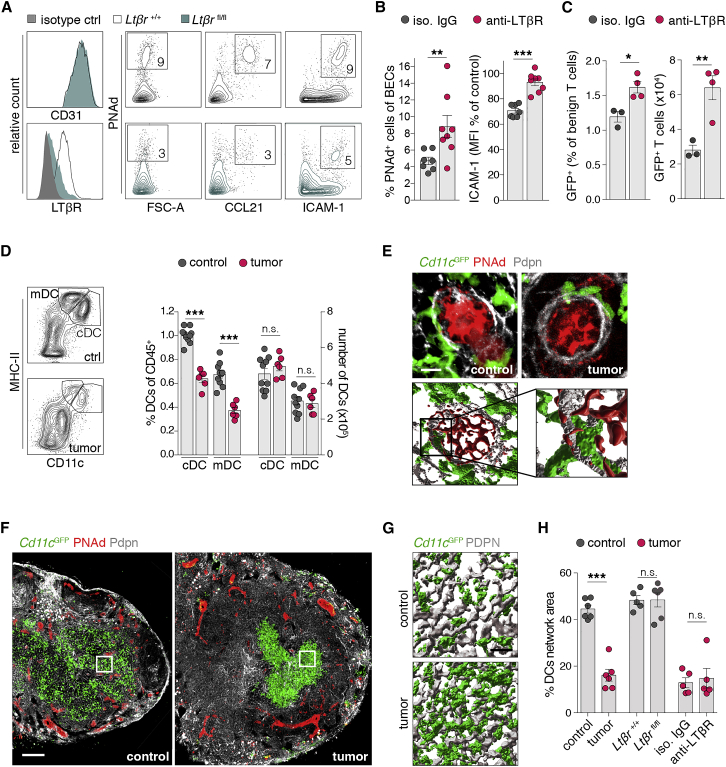
Loss of spatial vicinity of DCs and HEVs prohibits LTβR signaling (A) FCM analysis of surface molecules in HECs from LNs of *Ltbr*^+/+^ and *Cdh5*^CreERT2^-*Ltbr*^fl/fl^ mice (*Ltbr*^fl/fl^). Gating strategy given in Figure S5B. (B) Lymphoma-bearing mice treated with either isotype antibody or stimulatory anti-LTβR antibody. Quantitation of PNAd^+^ cells among BECs and ICAM-1 expression intensity in these cells as percentage of controls. n = 8 mice per group. (C) Quantification of LN infiltrating adoptively transferred lymphocytes by FCM. (D) Analysis of migratory DCs (mDCs) and classic DCs (cDCs). Left: proportion of DCs among all CD45^+^ cells. Right: total DC numbers per LN. Control n = 10, tumor n = 6 mice. (E) Top: confocal microscopy images of DCs in LNs of *Cd11c*^GFP^ mice with and without tumor. Bottom: segmented images to highlight cell-cell contacts. (F) Representative images of DC distribution in LN sections of *Cd11c*^GFP^ mice. Boxes indicate magnified and segmented areas. (G) Density of DCs (*Cd11c*^GFP^) among the FRC scaffold (PDPN). (H) Quantification of the proportion of the DC network area (determined by *Cd11c*^GFP^/MHC-II expression) relative to the area of the whole LN section in percent. n = 5–6 mice per group. Scale bars, 10 μm (E and G) and 100 μm (F). Mean and SEM are indicated. All data points represent individual animals. Statistics were calculated with Mann-Whitney U-test. ^∗^p < 0.05; ^∗∗^p < 0.01; ^∗∗∗^p < 0.005; n.s., not significant.

The maintenance of the HEC maturity requires constant LTβR signaling via recognition of membrane-bound LTα_1_β_2_ or LIGHT on dendritic cells (DCs) (Browning et al., 2005; Moussion and Girard, 2011). Conditional knockout of the LTβR in ECs (*Cdh5*^CreERT2^x*Ltbr*^fl/fl^ referred to as *Ltbr*^fl/fl^) caused a reduction of PNAd, CCL21, and ICAM1 expression in BECs (Figures 5A and S5A), similar to the dedifferentiation observed in HECs during lymphoma challenge. Treatment with a LTβR-stimulating antibody partially restored the occurrence of HECs (PNAd^+^ BECs) in tumor-bearing LNs and substantially improved immigration of naive T cells to a level similar as observed during control conditions (Figures 4B, 5B, and 5C).

LTα_1_β_2_ and LIGHT are differentially expressed in DCs, exhibiting a stronger expression in CD11c^high^MHC-II^medium^ classic DCs (cDCs), compared to CD11c^medium^MHC-II^high^ migratory DCs (mDCs) (Moussion and Girard, 2011). The proportion of both DC subsets relative to all CD45^+^ cells (including tumor cells) (Figure S5B) was reduced in tumor LNs compared to control conditions; however, the total numbers of cDCs and mDCs remained unchanged (Figure 5D). This indicated that DC persistence within, and immigration into, LNs was not affected by the tumor.

The expression of the LTβR in BECs and its ligands *Ltα*, *L*t*β*, and *T*nfsf*14* (LIGHT) in DCs remained unchanged in lymphoma (Figures S5C and S5D). DCs usually reside within the paracortex and the cortical ridge in the vicinity of HEVs, where they can encounter immigrating naive lymphocytes to facilitate effective T cell priming (Bajénoff et al., 2003; Mionnet et al., 2011). To our knowledge, a direct cell-cell contact of DCs and HEVs *in situ* has not yet been shown. We found DCs located in close proximity to HEVs and their appendages penetrating the vessel ensheathing FRC/pericyte (podoplanin, PDPN^+^) layer, enabling direct contact to HEVs. In tumor-challenged LNs, HEVs appeared to engage with lower numbers of DCs (Figure 5E). The DC network, which extended over the whole paracortex in control LNs, was markedly retracted during tumor conditions. The relocation resulted in an apparent increase of the distance between DCs and HEVs (Figure 5F). DC-occupied areas appeared condensed and exhibited a higher cell density; in contrast, in control LNs, DCs formed a widespread network (Figure 5G). Quantitatively, coverage of the LN area by the DC network was severely reduced in the tumor context. In contrast to published data (Simmons et al., 2019), positioning of DCs in LNs from *Ltbr*^fl^^/^^fl^ animals remained unchanged, indicating that the loss of HEV-derived CCL21 is dispensable for the migration of DCs within LNs. Systemic treatment with the LTβR-stimulating antibody failed to restore DC distribution (Figures 5H and S5E).

Collectively, these data reveal that signaling via the LTβR is required for HEC differentiation, while spatial separation between DCs and HEVs deprives HECs of such stimulation.

### Lymphoma growth disturbs CCL21 migration cues along the FRC network

Guided by CCR7, DCs constitutively migrate along CCL21 gradients expressed by and presented on the surfaces of fibroblastic reticular cells (FRCs) (Schumann et al., 2010). In LNs, DCs are essential for HEV integrity and lymphocyte homeostasis (Wendland et al., 2011). The B-cell-derived LTα_1_β_2_-FRC LTβR signaling axis regulates CCL21 expression in SLOs (Rehm et al., 2011; Chai et al., 2013). However, sensing lymph flow within the conduit system is an LN-specific prerequisite for CCL21 expression in FRCs (Tomei et al., 2009).

**Figure 6:**
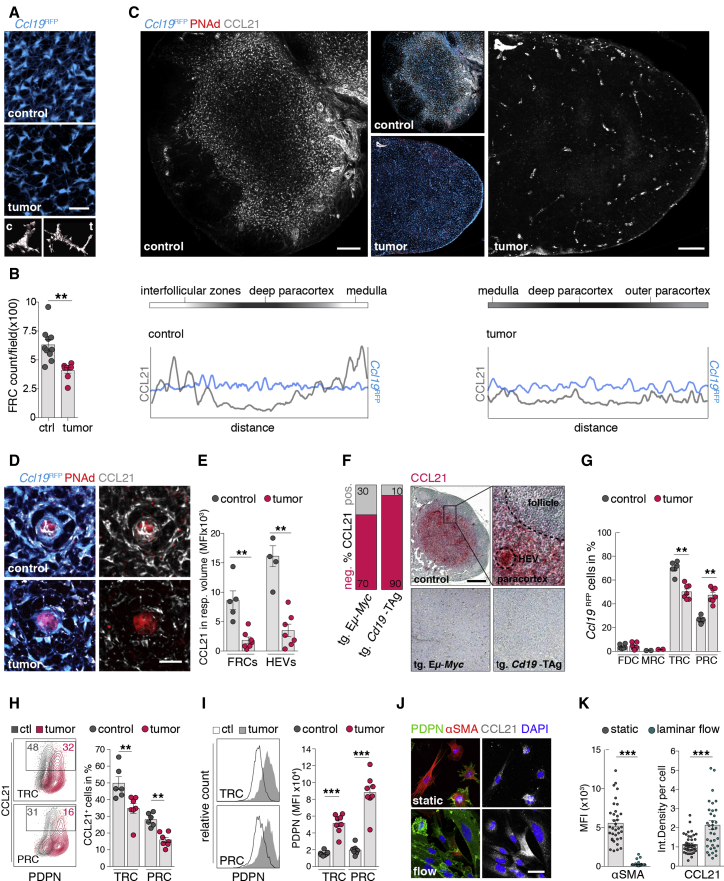
Lymphoma growth disrupts CCL21 migration cues on FRCs (A) Microscopic analysis of the FRC network (*Ccl19*^RFP^) in LN sections. Bottom: examples of FRC sphericity (*Ccl19*^RFP^ segmentation). (B) Quantification of the FRC density in LN sections of *Ccl19*^RFP^ mice. n = 7–10 mice per group. (C) Representative images of CCL21 expression in LNs sections of *Ccl19*^RFP^ mice and gradients of the fluorescence intensity over the distance. (D) CCL21 distribution around and within HEVs (PNAd^+^) and FRCs (*Ccl19*^RFP^). (E) Microscopic analysis of CCL21 (MFI) within masked *Ccl19*^RFP^ (FRCs) and PNAd^+^ (HEVs). Control n = 5, tumor n = 7. (F) Immunohistochemistry of CCL21 expression in controls and LNs from diseased tg. Eμ-*Myc* (n = 7) and *Cd19*-TAg mice (n = 11); box indicates magnified area. Bar graph depicts percentage of LNs with preserved (pos.) or lost (neg.) CCL21 expression. (G) Proportions of mesenchymal cell subsets (all *Ccl19*^RFP^), CD21/CD35^+^ follicular DCs (FDCs); MadCAM-1^+^ marginal reticular cells (MRCs); SCA-1^+^ pericytic reticular cells (PRCs); and T-zone reticular cells (TRCs). n = 4 mice per group. (H and I) FCM quantification of CCL21^+^ (n = 6–7 mice per group) (H) and PDPN^+^ expression (n = 7–8 mice per group) (I) in LNs of *Ccl19*^RFP^ mice. (J and K) Exemplary images (J) and analysis (K) of αSMA (MFI) and CCL21 (integrated density) in a FRC line cultured under static conditions or with laminar flow. Scale bars, 20 μm (A, D, and J) and 100 μm (C and F). Mean and SEM are indicated. Data points represent individual cells (K) or mice (B, E, and G–I). A Mann-Whitney U-test was applied. ^∗^p < 0.05; ^∗∗^p < 0.01; ^∗∗∗^p < 0.005.

In lymphoma-challenged LNs, the FRC network remained intact but featured a stretching of the network with larger and less spheroidic FRCs (Figures 6A and S6A). Although FRCs proliferated, their growth was less dynamic than those of Eμ-*Myc* tumor cells (Figure S6B) and resulted in a reduction of the FRC density within the paracortex (Figure 6B). Since *Ccr7* expression in DCs was preserved in tumors (Figure S6C), we examined FRC-derived CCL21, which formed a gradient from the deep paracortex toward the border and the cortical ridge in control LNs. This pattern disappeared in tumor-bearing LNs (Figures 6C and 6D). The CCL21 expression was strongly decreased in FRCs (*Ccl19*^RFP^) and HEVs (PNAd^+^) (Figure 6E). In controls, CCL21 was expressed in HEVs and throughout the paracortex but was largely absent in most LNs of diseased transgenic Eμ-*Myc* (70% of cases) and *Cd19*-TAg mice (90% of cases) (Figures 6F and S6E). To further analyze the functional consequences of deregulated CCL21 expression (Woolf et al., 2007), we employed intravital imaging of lymphocyte migration in popliteal LNs. The migration behavior of lymphocytes was significantly decreased in tumor-bearing LNs, indicating a disturbance of chemokine-controlled motility (Figure S6D). We also investigated different mesenchymal cell types with scaffolding and immunoregulatory functions to determine changes of the subset composition in tumors (Cheng et al., 2019; Krishnamurty and Turley, 2020) (Figures 6F and S6F). The proportions of follicular DCs (FDCs) and marginal zone reticular cells (MRCs) remained unchanged. T-zone reticular cells (TRCs) comprised the majority of FRCs in control LNs, but perivascular reticular cells (PRCs) were increased in tumor-bearing LNs (Figure 6G). TRCs and PRCs exhibited similar expression levels for CCL21 (Figure S6G), but it appeared that both subsets lost proportions of CCL21^+^ cells during tumor conditions (Figure 6H). The development of myofibroblasts into fully mature FRCs, including typical expression of PDPN and CCL21, requires LTβR signaling (Chai et al., 2013). In keeping with the results from Eμ-*Myc* B cells in mice, analysis of human B-NHL cell lines and DLBCL patient-derived xenograft (PDX) samples revealed modest gene expression of *LTβ*, compared with primary B cells (Figure S6H). We therefore asked if expanding lymphoma B cells act competitively to LTα_1_β_2_^high^-expressing normal B cells and, thus, cause insufficient LTβR stimulation in FRCs. Surprisingly, PDPN expression levels in FRCs under tumor conditions were, in fact, higher than in controls (Figure 6I). Moreover, overexpression of *Ltβ* in Eμ-*Myc* cells elicited higher levels of PDPN but failed to rescue FRC-derived CCL21 expression (Figure S6I). These data indicated a LTα_1_β_2_-LTβR signaling independent regulation of CCL21 in FRCs during tumor growth. On the other hand, a previous study showed that *in vivo* deprivation of afferent lymphatics resulted in a rapid ablation of *Ccl21* expression in FRCs (Tomei et al., 2009). Along this line, exposing a murine LN FRC line to shear stress as mediated by a laminar medium flow readily restored a more mature αSMA^low^PDPN^high^ phenotype and modest CCL21 expression (Figures 6J, 6K, and S6J).

Together, lymphoma-associated remodeling of the FRC network abrogated the CCL21 guidance track for immune cell trafficking within the LN parenchyma. This could likely be a result of deregulated CCL21 expression in FRCs or a secondary effect due to the physical disruption of the lymph architecture in rapidly expanding LNs during lymphoma progression.

### Tumor-induced LN expansion disrupts the conduit network

The differentiation state of FRCs and HEVs in LNs is critically dependent on afferent lymph flow as channeled by conduits within the reticular network of FRCs (Mebius et al., 1991; Tomei et al., 2009). B-cell-induced formation of follicles during development and LN expansion during immune responses disrupt conduit structures and abrogate lymph flow (Bajénoff and Germain, 2009; Martinez et al., 2019).

**Figure 7:**
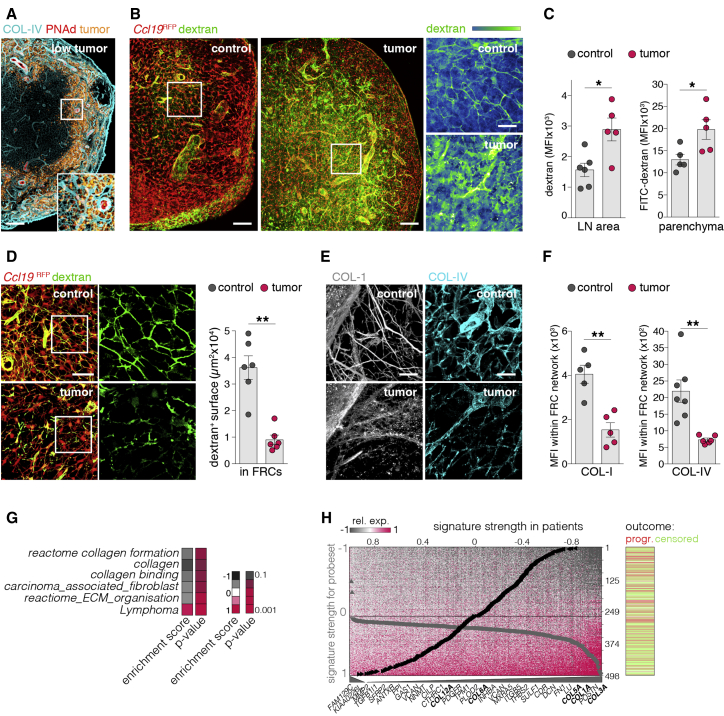
Lymphoma progression is accompanied by degeneration of the reticular conduit system (A) Representative LN section with low tumor (Eμ-*Myc* cells^CFP^), stained for PNAd and the basal lamina of the conduit system (COL-IV). (B) Representative images of FITC-dextran (10 kDa, sc injected) in draining LNs. (C) Quantification of FITC-dextran MFI in whole-LN sections and LN parenchyma (excluding template volume of the *Ccl19*^RFP^ FRC network). n = 5 mice per group. (D) Left: representative images of FITC-dextran (10 kDa, sc injected) within conduits (masked by the *Ccl19*^RFP^ FRC network). Right: quantification of FITC-dextran area within masked *Ccl19*^RFP^ FRC network. n = 6 mice per group. (E) Representative images of COL-IV and COL-I within the FRC network (*Ccl19*^RFP^ template). (F) Quantification of COL-IV and COL-I MFI within FRC network (*Ccl19*^RFP^ template). n = 5 mice per group. (G) Heatmap depicts enrichment scores (ESs) and p value (p) of the indicated Gene Ontology (GO) pathways. (H) DLBCL gene expression heterogeneity discovered by unsupervised signal dissection (SDCM). ECM and collagen-related genes are overrepresented, and signature expression is associated with patient’s outcome. All significantly correlated probe sets are depicted (462 correlated probe sets corresponding to 299 genes and 2 anti-correlated probe sets/genes from 54,675 measured in total). Right, outcome is depicted as progressive course (progr.) and non-progressive course (censored) for n = 498 cases. Boxes indicate magnified areas (A, B, and D). Scale bars, 100 μm (A and B) and 20 μm (D and E). Mean and SEM are indicated. All data points represent individual mice (C–F). All statistics were calculated with Mann-Whitney U-test. ^∗^p < 0.05; ^∗∗^p < 0.01.

We observed during early lymphoma progression that tumor cells relocated in the cortical ridge and at the border of the paracortex around HEVs, which are highly interconnected with the reticular conduit system (Figure 7A). We examined the integrity of the conduits and the afferent lymph flow by subcutaneous injection of fluorescently labeled dextrans (10 kDa). The transport of small molecules into the draining LNs was maintained; however, the molecules were not restricted to the conduit system but leaked into the parenchyma, indicating a loss of conduit integrity (Figures 7B and 7C). Moreover, a profound proportion of dextran-filled conduits was lost in LNs during tumor conditions (Figure 7D). Collagen-1 (COL-1) bundles as the main components of the conduit core, and collagen-IV (COL-IV) in the basal lamina of conduits (Sixt et al., 2005), appeared to be disrupted into discontinuous small fragments in LNs with tumor (Figure 7E). Using the FRC network (*Ccl19*^RFP^) as a mask (Figure S7A), we found a strong reduction of COL-IV and COL-I structures (Figure 7F), indicating a deregulation of FRC-derived extracellular matrix (ECM) components. Alternatively, tumor cells might actively degrade collagen fibrils. Although Eμ-*Myc* tumor cells expressed higher levels of the collagen-degrading matrix metallopeptidase (*Mmp)-14* than benign B cells, while FRCs upregulated *Mmp9* (Figure S7B), the application of broad spectrum MMP inhibitors did not impede the tumor-induced remodeling, as determined by the frequencies of PNAd^+^ BECs and CCL21^+^ FRCs in tumor-exposed LNs (Figure S7C). Several genes pivotal for the assembly of conduits, including collagens, laminins, and other ECM components, were downregulated in FRCs during tumor conditions (Figure S7D). Gene Ontology pathway analysis confirmed a negative enrichment of pathways associated with collagen formation, cancer fibrosis, and ECM organization (Figure 7G).

In line with our findings in the mouse model, negative enrichment of ECM and collagen-related genes (e.g., *COL3A*, *COL1A*, *COL5A*, *COL12A*) was significantly overrepresented (p = 1.9 × 10^−14^, hypergeometric test) within a signature of strong systematic heterogeneity that was discovered by unsupervised signal dissection (SDCM) of gene expression in human DLBCL (Grau et al., 2019) (Figure 7H). Association of this signature’s average expression with patient outcome showed that reduced collagen expression is significantly associated with a progressive course of the disease (p = 9.6 × 10^−4^) (Figure S7E).

Collectively, we here not only linked a restricted conduit channeled lymph flow in aggressive BCL with a deteriorated ECM deposition, but also revealed the consequences for the differentiation and functional capacity of HEVs.

## Discussion

In this study, we dissect the dichotomy of angiogenesis in aggressive BCL, characterized by an increase in microvessel density on the one hand and loss of mature HEVs on the other. We provide functional evidence that not only is the multistep dedifferentiation program of HEVs tightly synchronized with the conduit-mediated lymph flow, but LN remodeling in lymphoma also leads to impaired T lymphocyte transmigration. These processes shape a disturbed LN microarchitecture in which spatially organized interactions among patrolling T lymphocytes, FRC guidance cues, and antigen-presenting DCs required for mounting productive anti-tumor immune responses are diminished.

Diseased Eμ-*Myc*, *Cd19-Tag*, and lymphoma-transplanted mice phenocopy vascular alterations of human aggressive B-NHL. In addition to enhanced MVD (Gloger et al., 2020), both feature a severe loss of HEV structures. Hallmarks of a gradual dedifferentiation of HECs correlating with tumor progression included reduced expression of PNAd, ICAM-1, CCL21, and the ectoenzyme ATX. Loss of these HEV-associated factors severely impaired the transendothelial migration of naive lymphocytes *in vivo*, which is consistent with studies where HEVs were experimentally deprived of LTβR signaling (Browning et al., 2005; Onder et al., 2013) or exposed to pharmacological ATX inhibition (Kanda et al., 2008; Bai et al., 2013).

Here, we report that the coadministration of T cells and the ATX product, LPA, enhanced T cell transendothelial migration into the LN parenchyma through residual HEVs, but LPA could not fully compensate for the loss of adhesion molecule and chemokine expression that is crucial for the rolling and adhesion of lymphocytes (Girard et al., 2012). In lymphoma-challenged LNs, antibody-mediated LTβR stimulation was sufficient to restore HECs and their functional surface molecule repertoire. An effort to restore CCL21 expression in FRCs, as mediated by LTβR antibody stimulation or overexpression of LTβR ligands in lymphoma cells, was unsuccessful, indicating that FRCs require mechanosensitive signaling to gain a mature myofibroblastic state.

In addition, a severe disturbance of the ECM components COL-I and IV, which bind and locally arrange CCL21 (Yang et al., 2007), could cause the decay of the intranodal chemokine gradient, visible as an altered lymphocyte motility. Lower CCL21 expression in FRCs was closely linked to an aberrantly contracted DC network distant from HEV structures, similar to LNs of CCL21 knockout mice (Link et al., 2007). Functionally, loss of a DC-HEV contact zone (Bajénoff et al., 2003; Moussion and Girard, 2011) may impair anti-tumor priming facilitated by a naive T lymphocyte-DC encounter. In support of this view, we previously showed that in Eμ-*Myc* lymphoma-bearing mice, DCs failed to efficiently prime and activate T cells (Rehm et al., 2014); however, a role of the FRCs and conduit system was not explored in that study.

FRC differentiation is regulated by LTβR signaling as well; they are crucially involved in the zonal organization of LNs and control of immune cell interactions therein (Chai et al., 2013). In lymphoma, such a signal is missing, as conduit deconstruction hinders lymph flow within the reticular system. In contrast to infection models (Liao and Ruddle, 2006; Tzeng et al., 2010; Dasoveanu et al., 2016), lymphoma did not allow a recovery of HEVs, FRC-derived CCL21 expression, or the conduit network, although lymphoma B cells potentially can activate LTβR signaling (Rehm et al., 2011; Gloger et al., 2020). Notably, LN FRCs are different from splenic FRCs, as they require for their differentiation a dual stimulation of the LTβR and a cooperative mechanical stimulus provided by the lymph flow within the reticular conduit system (Tomei et al., 2009). Hence, we suggest that a cooperation of LTβR signaling and a mechanical stimulus is necessary for stroma reconstruction.

Microscopic examination revealed a damaged HEV morphology in high-grade lymphomas, while in low-grade lymphomas, HEVs were preserved (Pajor et al., 1990). In line, we found a strong reduction and even entire loss of HEVs in DLBCL and BL, a finding that was in strong contrast to largely intact HEVs in cHL. Different from the two aggressive B-NHL entities, cHL harbors only a minority of tumor-defining Hodgkin-Reed-Sternberg (HRS) cells, surrounded by a predominant benign immune cell infiltrate (Menzel et al., 2020). Data showed that HRS-derived LTα activates ECs to enhance naive T cell recruitment by upregulating adhesion molecules and the ECM component hyaluronan (Fhu et al., 2014). The capacity to recruit naive and central memory T lymphocytes via the CCR7-CCL21 signaling axis can likely be attributed to intact HEVs, which, we envision, serve as a prerequisite for the clinical benefit of immune checkpoint blockade (ICB) in cHL (Alencar and Moskowitz, 2019). Apart from HEV-unrelated differences between cHL and DLBCL pathology, it is tempting to correlate a potential role of mature HEVs in efficient ICB because clinical trials of programmed cell death protein **1 (**PD-1) inhibition in DLBCL did not show clinical efficacy (Ansell et al., 2019; Kline et al., 2020).

In sum, our data suggest that HEV dedifferentiation is caused by a lymphoma-induced cascade that begins with an abrogation of lymph flow in conduits and a downregulation of FRC-derived CCL21 expression. Disordered CCL21 distribution in the context of a disturbed ECM causes a disturbance of lymphocyte and DC migration cues, causing a loss of spatial proximity between HEVs and DCs, which impairs homeostatic signaling of the LTβR in HEVs. Functionally, the failure of dedifferentiated HEVs to bind T lymphocytes results in a transmigration deficit and, ultimately, establishes a lymphoma survival niche not monitored by patrolling T lymphocytes.

### Limitations of the study

The present study describes a complex regulatory and functional interaction of LN stromal cells, malignant B cells, and LN-resident immune cells. Experimental results were generated in the Eμ-*Myc* mouse lymphoma model and further confirmed in an SV40 large T-antigen-driven aggressive lymphoma model. Although mouse models cannot exactly phenocopy B cell origin, phenotype, and genetics of defined human lymphoma entities, they are suitable to mimic features like growth kinetics, homing, migration, and dependency on a non-malignant LN infrastructure. For example, Eμ-*Myc* tumor cells arise from pre-B cells in the bone marrow, in contrast to the human germinal center (GC) and post-GC lymphomas used in this study for comparison. Furthermore, the Eμ-*Myc* model can provide insight into processes at tumor onset in an *in vivo* setting, whereas human specimens are usually obtained from established and progressed lymphomas. The *Myc*-driven aggressive mouse lymphoma model provided conclusive data on the interpretation of lymphoma pathology in a microenvironmental context. For validation in human specimens, the lymphoma entities DLBCL and Burkitt’s lymphoma were chosen, which share an aggressive growth kinetics with the Eμ-*Myc* mouse model. Although HEV dedifferentiation was identified as a major cause of insufficient T lymphocyte immigration and, thus, loss of immunosurveillance in lymphoma, the study could not fully solve a repair mechanism suitable for a putative translational approach in immunotherapy. Potentially, immature endothelial precursors could contribute to the loss of HEV structures.

Considering the limitations of mouse models, future dissection of the lymphoma-stroma interface will profit from high-resolution scRNA-seq analysis in patients. Functional and even local interactions between tumor and stromal cells can be inferred from this type of analysis. However, we suggest that for deeper kinetic and mechanistic validation of scRNA-seq-derived datasets, mouse models remain essential.

## STAR★Methods

### Key resources table

**Table:** 

REAGENT or RESOURCE	SOURCE	IDENTIFIER
**Antibodies**		

CD31 (clone: 390, BV785)	BioLegend	Cat#102435; RRID:AB_2810334
CD45 (clone: 30-F11, AF700)	BioLegend	Cat#103127; RRID: AB_493714
TER119 (clone: Ter-119, AF700)	BioLegend	Cat#116220; RRID: AB_528963
PDPN (clone: 8.1.1, PE-Cy7)	BioLegend	Cat#127411; RRID: AB_10613648
CD36 (clone: HM36, PerCP-Cy5.5)	BioLegend	Cat#102619; RRID: AB_2750187
CXCR4 (clone: L276F12, PE)	BioLegend	Cat#146505; RRID: AB_2562782
JAG-1 (clone: HMJ1-29, APC)	BioLegend	Cat#130913; RRID: AB_2561304
PNAd (clone: MECA-79, AF647)	BioLegend	Cat#120801; RRID: AB_493555
PNAd (clone: HECA-452, AF594)	BioLegend	Cat#321317; RRID: AB_2734303
ICAM-1 (clone: YN1/1.7.4, APC-Cy7)	BioLegend	Cat#116125; RRID: AB_2716073
VCAM-1 (clone: 429, Pacific Blue)	BioLegend	Cat#105722; RRID: AB_2304290
CCL21 (clone: AF457, AF488)	R&D	#AF457-SP; RRID: AB_2072083
CD3 (clone: 17A2, BV510)	BioLegend	Cat#100233; RRID: AB_2561387
CD4 (clone: GK1.5, Spark Blue550)	BioLegend	Cat#100473; RRID: AB_2819768
CD8 (clone: 53-6.7, BV421)	BioLegend	Cat#100737; RRID: AB_10897101
CD62L (clone: MEL-14, FITC)	BioLegend	Cat#104405; RRID: AB_313092
CCR7 (clone: 4B12, PE-Cy7)	BioLegend	Cat#120123; RRID: AB_2890830
MadCAM-1 (clone: MECA-367, AF488)	BioLegend	Cat#120707; RRID: AB_493399
CD21/35 (clone: 7E9, Pacific Blue)	BioLegend	Cat#123413; RRID: AB_2085159
LTbR (clone: 5G11, PE and purified)	BioLegend	Cat#134403; RRID: AB_1659180
αSMA (clone: 1A4, Cy3)	Sigma Aldrich	Cat#C6198; RRID: AB_476856
CD16/CD32 (clone: 93, purified)	BioLegend	Cat#101301; RRID: AB_312800
LYVE-1 (clone: ALY7, purified)	eBioscience	Cat#14-0443-82; RRID: AB_1633414
Rat IgG Isotype control (clone: RTK2758, purified)	BioLegend	Cat#400544; RRID: AB_11147167
Collagen-IV (ab19808, purified)	Abcam	Cat# ab19808; RRID: AB_445160
Collagen-I (purified)	BioRad	Ref.2150-1410; RRID: AB_620433
Autotaxin/ENPP2 (purified)	Bioss	Cat# bs-6279R; RRID: AB_11118821
Alexa- Fluor-488/568/647 conjugated secondary antibodies	ThermoFischer	N/A
CD3 (clone: 145-2C11, Ultra_LEAF, purified)	BioLegend	Cat#100339; RRID: AB_11150783
CD28 (clone: 37.51, Ultra_LEAF, purified)	BioLegend	Cat#102115; RRID: AB_11150408
CD62L (clone: MEL-14, FITC)	BioLegend	Cat#104405; RRID: AB_313092
CD44 (clone: IM7, APC)	BioLegend	Cat#103011; RRID: AB_312962
Vα2 (clone: B20.1, AF488)	BioLegend	Cat#127819; RRID: AB_2687229
MHC II (clone: 10-3.6, PE)	BioLegend	Cat#109908; RRID: AB_313457
CD3 (clone: 17A2, BV495)	BioLegend	Cat#100240; RRID: AB_2563427
CD11c (N418, Pacific Blue)	BioLegend	Cat#117321; RRID: AB_755987
B220 (RA3-6B2, APC)	BioLegend	Cat#103211; RRID: AB_312996
CD4 (SK3, Spark Blue550)	BioLegend	Cat#344655; RRID: AB_2819978
CD19 (6D5, APC/cy7)	BioLegend	Cat#115529; RRID: AB_830706
GFP (purified)	ThermoFischer	Cat#A-11122; RRID: AB_221569

**Bacterial and virus strains**		

*E. coli* XL1 Blue	Internal stock	N/A

**Biological samples**		

DLBCL Multiple tissue array	US Biomax	OD-CT-LyMly02-001
Hodgkin’s lymphoma Multiple tissue array	US Biomax	HL481b
Different lymphoma entities Multiple tissue array	US Biomax	LY2081a

**Chemicals, peptides, and recombinant proteins**		

Marimastat	Selleckchem	Cat#S7156
Ilomastat	Selleckchem	Cat#S7157
NSC405020	Selleckchem	Cat#8072
7AAD	BioLegend	Cat#420403
Tamoxifen	Sigma Aldrich	Cat#T5648
GlutaMAX	ThermoFischer	Cat#35050061
Penicillin/Streptomycin	ThermoFischer	Cat#15140122
murine IL-7	Peprotech	Cat#217-17
murine IL-15	Peprotech	Cat#210-15
Lipopolysaccharide	Sigma Aldrich	Cat#L2630
CD45 Microbeads	Miltenyi	Cat#130-052-301
Ovalbumin	Sigma Aldrich	Cat#A5503
Histofix	Carl Roth	Cat#P087.1
DNase1	ThermoFischer	Cat#EN0521
Dispase-II	Roche	Ca#04942078001
Collagenase-P	Roche	Ca#CollP-RO
Toluol	Carl Roth	Cat#KK46.1
SHIELD Tissue Preservation	Lifecanvas technology	N/A
Sucrose	Sigma Aldrich	Cat#S0389
Quadrol	Sigma Aldrich	Cat#122262
Triethanolamine	Wako	Cat#145-05605
Urea	Sigma Aldrich	Cat#U5378
Triton X-100	Sigma Aldrich	Cat#T8787
Mineral oil	Sigma Aldrich	Cat#M8410
Xylol	Sigma Aldrich	Cat#214736
Biotin Blocking System	Dako	Cat#X0590
Alkaline Phosphatase	Dako	Cat#K5005
Pierce Horeseradish Peroxidase	Thermo Scientific	Cat#31490
AEC substrate-chromogen	Dako	Cat#K346111-2
Hematoxylin	Fisher Scientific	Cat#H345-25
Kaiser’s glycerol gelatin	Carl Roth	Cat#6474.1
SuperScript VILO cDNA Synthesis kit	Thermo Scientific	Cat#11754050
TaqMan Gene Expression Master Mix	Applied Biosystems	Cat#4369016
RPMI-1640 Medium	GIBCO	Cat #11530586
DMEM Medium	GIBCO	Cat #11995073
Fetal Bovine Serum	GIBCO	Cat #26140079
Fetal Bovine Serum fatty acid free	Sigma Aldrich	Cat#A6003
Sodium pyruvate (100x)	GIBCO	Cat #12539059
2-Mercaptoethanol	Thermo Fisher	Cat #11528926
Bovine Serum Albumin	Sigma Aldrich	Cat #05470
Dulbecco’s Phosphat buffered Saline	Biowest	Cat#P0750
Fluoresceinisothiocyanat dextran wt10,000	Sigma Aldrich	Cat#FD10S
4’,6-Diamidino-2-phenyl-indol-dihydrochlorid	Sigma Aldrich	Cat#D9542
Corn oil	Sigma Aldrich	Cat#C8267
Agarose low melt	Carl Roth	Cat#6351.5
Immu-Mount Shandon	Thermo Scientific	Cat#9990402
Oleoyl-L-a-lysophosphatidic acid	Sigma Aldrich	Cat#L7260
Isoflurane	cp-pharma	Cat#1214
RetroNectin	TaKaRa	Cat#T110A

**Critical commercial assays**		

Click-iT EdU assay	Thermo Fisher	Cat#C10337
Fix & Perm Cell Permeabilization Kit	Thermo Fisher	Cat#GAS003
RNeasy Mini Kit	QIAGEN	Cat #74104
mouse *Ltb* TaqMan Primer	Thermo Fisher	Mm00434774_g1
mouse *Actb* TaqMan Primer	Thermo Fisher	Mm00607939_s1
human *LTB* TaqMan Primer	Thermo Fisher	Hs00242739_m1
human *B2M* TaqMan Primer	Thermo Fisher	Hs00187842_m1

**Deposited data**		

scRNA-seq	this paper	ArrayExpress: E-MTAB-10389
Microarray data	Gloger M. et al., 2020	GEO repository: GSE126033
Microarray data	Grau M. et al., 2019	GEO repository: GSE31312

**Experimental models: Cell lines**		

DOHH-2	DSMZ	Cat# ACC 47
SU-DHL-4	DSMZ	Cat# ACC 495
OCI-Ly7	DSMZ	Cat# AC 688
JEKO-1	DSMZ	Cat# ACC 553
JURKAT	DSMZ	Cat# ACC 282
RAJI	DSMZ	Cat# ACC 319
PlatE	Cell Biolabs	Cat# RV-101
murine lymph node FRCs	Sophie E. Acton; Univ. College London, London, UK	https://doi.org/10.1038/nature13814
RAJI	DSMZ	Cat# ACC 319

**Experimental models: Organisms/strains**		

C57BL/6N mice	Charles River	Strain Code: 027
Tg(Cdh5-Cre/ERT2)1Rha56 mice	R. Adams; Max-Planck-Institute, Münster, Germany	N/A
Ubow7,57 mice	M. Bajenoff; Aix Marseille Univ., INSERM, CNRS, Marseille, France	N/A
Ccl19-Cre mice	B. Ludewig; Kantonsspital St. Gallen, St. Gallen, Switzerland	N/A
Cd11c-Cre mice	Jackson Laboratories	JAX: 007567
Ltbr-fl/fl (Ltbrtm1.1thhe) mice	T. Hehlgans; RCI Regensburg, Regensburg, Germany	N/A
B6.Cg-Tg (IghMyc)22Bri/J mice	Jackson Laboratories	JAX: 002728
Ubc-GFP mice	Jackson Laboratories	JAX: 004353

**Oligonucleotides**		

CpG-ODN1668	InvivoGen	Cat#tlrl-1668

**Software and algorithms**		

Imaris (v.9.7.2)	Bitplain	N/A
Arivis Vision4D x64 (v.3.4.0)	Arivis	N/A
GraphPad Prism (v.7)	GraphPad	https://www.graphpad.com:443/
FlowJo (v.10.6.1)	BD	https://www.flowjo.com/solutions/flowjo
ImageJ (v.2.1.0)	National Institutes of Health, USA	https://imagej.nih.gov/ij/
RStudio (v.1.2.5042)	CRAN R-Project	https://www.R-project.org
Seurat (v.2.1)	CRAN R-Project	https://satijalab.org/seurat/
Monocle (v.2)	Bioconductor	https://www.bioconductor.org/packages/release/bioc/html/monocle.html
STARsolo (v.2.7.3)	GitHub	https://github.com/alexdobin/STAR/blob/master/docs/STARsolo.md
scevelo (v.0.2.3)	PyPI	https://scvelo.readthedocs.io/about/
Excel (v.14.0.0)	Microsoft	N/A
Adobe Illustrator CS6	Adobe	N/A
Adobe Photoshop CS6	Adobe	N/A
Zen blue edition	Carl Zeiss	N/A
Zen black edition	Carl Zeiss	N/A

### Resource availability

#### Lead contact

Further information and requests for resources and reagents should be directed to and will be fulfilled by the Lead Contact, Armin Rehm (arehm@mdc-berlin.de).

#### Materials availability

Key resources including details of key reagents and cell lines used are available in the Key resources table. All unique/stable reagents generated in this study are available from the Lead Contact with a completed Materials Transfer Agreement.

### Experimental model and subject details

#### Animals

C57BL/6N (Charles River), Cdh5-CreERT2 (Tg(Cdh5-Cre/ERT2)1Rha (Wang et al., 2013), Ubow (Ghigo et al., 2013; Mondor et al., 2016); Ccl19-Cre (kindly provided by B. Ludewig)(Ludewig et al., 2012; Chai et al., 2013), Cd11c-Cre (Jackson Laboratories), Ubc-GFP (Jackson Laboratories), OT-II (Jackson Laboratories), Ltbr-fl/fl (Ltbrtm1.1thhe, kindly provided by T. Hehlgans)(Wimmer et al., 2012). Transgenic mice were all backcrossed onto a C57BL/6N background. All mice were bred and maintained in a pathogen-free environment at the animal core facility of the Max-Delbrück-Center for Molecular Medicine Berlin, Germany. Light cycles were at 12 hr intervals, temperature was kept at 22°C, and humidity at 55%, in compliance with the institutional rules. Male and female mice between the age of 8-12 weeks were used in all experiments. Experimental groups were sex-matched according to the origin of the donor Eμ-*Myc* clone. All experiments were conducted in compliance with the institutional guidelines of the Max-Delbrück-Center for Molecular Medicine and approved by the Landesamt für Gesundheit und Soziales Berlin, Germany (G0104/16; G0052/12; G0373/13; G0058/19; G0044/16).

#### Human and murine cell lines and primary cells from normal tissue

The human B-NHL cell lines DOHH-2 (ACC 47, follicular lymphoma), SU-DHL-4 (ACC 495) and OCI-Ly7 (ACC 688; both diffuse large B cell lymphoma, DLBCL), JeKo-1 (ACC553; mantle cell lymphoma, MCL), Raji (ACC 319; Burkitt lymphoma, BL), and the Jurkat cell line (ACC 282, acute lymphoblastic leukemia, T-ALL) were purchased from DSMZ (Braunschweig, Germany). Upon receipt, cell lines were expanded and stored as early passage frozen aliquots. Patient-derived xenograft (PDX) samples from DLBCL cases that were passaged via NSG mice were obtained from Dana-Farber Cancer Institute (PRoXe Depository, Boston, MA). All cell lines and PDX samples were authenticated by the commercial providers. Flow cytometry was used to verify human origin and B- or T cell lineage derivation, according to the phenotypes provided by the repository. The sex of the cell lines is given by the repository (DSMZ). Primary B cells (7AAD^-^CD45^+^CD69^-^CD4^-^CD8^-^CD14^-^CD19^+^) and primary T cells (7AAD^-^CD45^+^CD69^-^CD19^-^CD14^-^CD3^+^) from healthy male and female donors were purified from PBMCs via a Ficoll gradient and further sorted by flow cytometry.

A murine LN FRC cell line (mFRC) was generated as previously described (Acton et al., 2014). These cells were maintained in DMEM medium with high glucose, 10% fetal calf serum, 1% penicillin/streptomycin, and 1% insulin-transferrin-selenium (all ThermoFisher Scientific) at 37°C, and 5% CO_2_. For static conditions, mFRCs were cultured on poly-L-lysine coated (0.01% in H_2_O) glass slides at a density of 4x10^5^ cells/ml for 24 hours. Under conditions of laminar flow, mFRCs were cultured on Luer channel slides (Ibidi) with 4x10^5^ cells/ml, and laminar flow with culture medium was applied using a micro-pump (flow rate 0.5 ml/min) for 24 hours, as described previously (Tomei et al., 2009).

#### Human tissue specimen

Multiple tissue arrays (MTA, all from US Biomax) contained various specimens of DLBCL (Nr. OD-CT-LyMly02-001; core diameter 2 mm; 30 cases) or classical Hodgkin’s lymphoma (Nr. HL481b; core diameter 1.5 mm, 46 cases) or different lymphoma entities e.g., DLBCL (49 cases), Burkitt’s lymphoma (2 cases), and follicular lymphoma (3 cases), (Nr. LY2081a, core diameter 1 mm). All samples were not further diagnosed according to cytogenetic rearrangements. MTAs contained tissue specimen from male and female patients, according to the information provided by the vendor. IHC was performed as described below. The study involving primary human tissues was conducted according to the declaration of Helsinki and in accordance with ethical guidelines and votes provided by the manufacturer.

### Method details

#### Retroviral transduction of Eμ-Myc tumor cells

Retroviral particles were generated by transient co-transfection of Platinum-E cells with the MP71 vector, containing either a *mTurquoise2* (referred to as CFP) or *Ltb-P2A-mScarlet* transgene cassette. Viral transduction was performed in non-tissue-culture treated plates coated with 12,5 μg/ml RetroNectin (TaKaRa). To ensure efficient transduction Eμ-*Myc* B cells were thawed 24 hours pre-transduction and activated using 10 μg/ml lipopolysaccharide. 5 × 10^5^ Eμ-*Myc* cells (one clone per construct) per ml of virus supernatant supplemented with protamine sulfate were spinoculated at 800 × *g* for 90 min at 32 °C. Fluorescent reporter^high^ transduced cells were enriched by FACSAria Fusion cell sorter (BD), expanded and stored in liquid nitrogen. For *Ltb-P2A-mScarlet* Eμ-*Myc* cells, reporter^negative^ cells were sorted, expanded and stored concomitantly with the reporter^high^ cells to enable tumor transfer experiments with a similarly processed clone.

#### Tumor cell transfer

3x10^5^ Eμ-*Myc* tumor B cells were transferred intravenously (i.v.) in RPMI-1640 medium into recipient mice; at least 2 to 6 independent lymphoma clones derived from Eμ-*Myc* mice were tested for each animal experiment. Tumor load in LNs of recipient mice was determined by flow cytometric analysis (CD45^+^B220^med^FSC-A^high^).

#### Spontaneous tumors in trangenic mice

Transgenic Eμ-*Myc* mice (B6.Cg-Tg (IghMyc)22Bri/J) and *Cd19*^Cre^xTAg mice (Hoser et al., 2018) (all C57BL/6 background) sporadically developed tumors with nodal involvement. All mice with palpable LNs or other signs of distress were sacrificed to dissect LNs. Tissue samples were formalin-fixed and embedded in paraffin (Hoser et al., 2018).

#### Tamoxifen treatment

Tamoxifen was dissolved in corn oil and applied by gavage. *Cdh5*^CreERT2^xUbow mice were treated with Tamoxifen on 2 consecutive days ending 14 days before tumor transfer. Reporter mouse strains were treated on 4 consecutive days ending 3 days before analysis. *Cdh5*^CreERT2^x*Ltbr*^fl/fl^ mice were treated with Tamoxifen on 4 consecutive days until 7 days prior to analysis.

#### Adoptive lymphocyte transfer

Lymphocytes were isolated from LNs and spleen of *Ubc*-GFP mice. For LPA treatment, 20 μg LPA (Sigma, 0.1% BSA in PBS) was added to the cell suspension immediately before transfer. For transfer of activated T cells, 2 × 10^6^ cells/ml in RPMI-1640, 10% FCS, 1% Penicillin/Streptomycin, 1% Glutamax, 1% essential amino acids, 50 ng/ml IL-15 and 10 ng/ml IL-7 (both Peprotech) were activated with plate bound 1 μg/ml anti-CD3 mAb, 0.1 μg/ml anti-CD28 mAb (both BioLegend) and expanded for 7 days until transfer. 3 × 10^7^ cells (in RPMI-1640) were injected i.v. into recipient mice. 90 minutes after cell transfer, mice were sacrificed, LNs were excised, and cells were analyzed by flow cytometry and immunohistochemistry.

#### Antigen-specific T cell activation *in vivo*

CD4^+^ cells from spleen and LNs of OT-II transgenic mice were enriched by negative selection applying magnetic cell sorting as to the manufacturer’s instructions (Miltenyi). 1x10^6^ CD4^+^ were transferred (i.v.) into control or Eμ-*Myc* tumor challenged mice at day 9 of tumor challenge. Mice were immunized with ovalbumin (Sigma-Aldrich; 100 μg) and CpG-ODN1668 (BioTez Berlin; 25 μg) five hours after CD4^+^ OT-II cell transfer. Cell count and activation status were determined in draining lymph nodes by flow cytometry.

#### *In vivo* proliferation assay

Mice were i.p. injected with EdU solution (1 mg in 0.9% NaCl) on 3 consecutive days, starting 4 days prior to analysis. LN stroma cell suspensions were prepared and analyzed by flow cytometry using a Click-iT EdU assay according to the manufacturer’s instructions (Thermo Fisher).

#### Antibody and pharmacological treatment

Matrix metalloproteinase (MMP) inhibitors Marimastat, Ilomastat, and NSC405020 (150 mg/kg/b.w. in PBS, (all Selleckchem) and LTβR-stimulating antibody (15 μg, clone 5G11, BioLegend) or IgG1 isotype control antibody (clone: RTK2758) were intraperitoneally injected on 4 consecutive days prior to analysis.

#### *In vivo* conduit-filling

Nine to ten days after tumor cell transfer, *Ccl19*^RFP^ reporter mice were treated by footpad injection with 10 μL of FITC-dextran solution (10 kDa, 100 mg/ml, Sigma Aldrich). Mice were sacrificed after 3 min, popliteal LNs were collected, fixed in 4% paraformaldehyde (PFA) solution (Carl Roth) overnight (ON) and processed for microscopic analysis.

#### Isolation of BECs from murine lymph nodes

Cervical, axillary, brachial and inguinal LNs were dissected and enzymatically digested in RPMI-1640 medium containing 0.8 mg/ml Dispase-II, 0.2 mg/ml Collagenase-P (both Roche) and 0.1 mg/ml DNase1 (ThermoFisher) for a maximum of 40 min at 37°C under frequent tissue disruption by pipetting and transfer of detached cells to ice cold FBS (30% in PBS). The stromal cell fraction was enriched by leukocyte depletion using anti-CD45 magnetic cell sorting (Miltenyi), essentially as described (Gloger et al., 2020).

#### Flow cytometry and fluorescence associated cell sorting

Erythrocytes in cell suspensions were depleted using erythrocyte lysis buffer (9 mg/ml NH_4_Cl, 1 mg/ml KHCO_3_, 10 mM EDTA in ddH_2_O) for 5 min on ice. Cells were blocked with anti-CD16/CD32 antibody, followed by antibody staining in flow cytometry buffer (5% FBS, 2 mM EDTA in PBS) for 15 min at 4°C. Intracellular staining was performed with the Fix & Perm Cell Permeabilization Kit (ThermoFisher Scientific). Dead cells were detected with 7AAD (BioLegend). All cells were analyzed on an Aurora spectral cytometer (Cytek), or a FACSCanto II instrument (BD Bioscience). Data were further analyzed with FlowJo software (v.10, TreeStar).

For single-cell RNA sequencing, ∼6x10^4^ BECs (7AAD^-^CD45^-^TER119^-^PDPN^-^CD31^+^) were sorted into HBSS buffer (4°C) using a FACS Aria Fusion instrument (100 μm nozzle; ∼8x10^3^ events/s flow rate). Freshly sorted cells were centrifuged at 400 *g* for 5 min and resuspended in 30 μl of HBSS. The cell count was determined using a Neubauer chamber and adjusted to 500 viable cells per μl.

#### Single-cell RNA sequencing

The cell suspensions containing sorted BECs were processed for single-cell RNA sequencing (scRNA-seq) using the Chromium Single Cell 3′ library and Gel Bead Kit v2 (10X Genomics) according to the manufacturer’s guidelines. Libraries were sequenced on an Illumina NextSeq 500 using 150 cycles high output V2 kit. The Cell Ranger package (v.3.0.2) was used to align high quality reads to the mm10 transcriptome. Single cells from tumor and control samples were integrated using the FindIntegrationAnchors function from the Seurat package with the default parameters, as implemented in the CellRanger (v2.1.0) pipeline. Data analysis and generation of representative plots was performed with the Seurat (v.2.1) package in R (Stuart et al., 2019). Supervised cell selection of scRNA-seq data was used to remove non-BECs according to subpopulation specific marker genes: BECs (*Cdh5*^+^, *Pecam1*^+^), leukocytes (*Ptprc*^*-*^*, Cd52*^*-*^), lymphatic endothelial cells (*Prox1*^*-*^*, Lyve1*^*-*^*, Pdpn*^*-*^), mesenchymal cells (*Acta2*^*-*^*, Pdpn*^*-*^*, Pdgfb*^*-*^). Cells were attributed to blood endothelial subpopulations based on canonical marker expression: all (*Pecam1*^+^), art (*Sox17*^+^, *Gja4*^+^, *Rbp7*^+^), vn (*Lrg1*^+^, *Vwf*^+^, *Il6st*^+^, *Chst4*^-^), HECs (*Glycam1*^*+*^, *Chst4*^*+*^), cap_vn (*Enpp2*^+^, *Col4a1*^+^, *Aplnr*^+^), cap_art (*Rgcc*^+^, *Ly6c1*^+^, *Ramp3*^+^), tip cells (*Esm1*^*+*^*, Cxcr4*^*+*^), stalk cells (*Jag1*^*+*^, *Sdpr*^*+*^), quiescent cells (*Cd36*^*+*^, *Flt1*^*+*^, *Mki67*^-^, *Cdk1*^-^)(Zhao et al., 2018; Brulois et al., 2020; Kalucka et al., 2020). For Single-cell trajectories, RNA for pseudotime analysis were computed using the Monocle package (v.2). For RNA velocity, spliced and unspliced reads were quantified using STARsolo (v.2.7.3) and analysis was performed using mapped reads with scevelo 10.1038/s41587-020-0591-3 (v.0.2.3). GSEA was performed on the results of pairwise comparison between HEC cluster HEC_1_ and HEC_3_. Briefly, genes were ranked according to their significance and cluster association by multiplying the log value of the adjusted p value from Seurat’s FindMarkers function by the sign of the associated fold change for that gene. Gene sets from The Molecular Signatures Database hallmark gene set collection (curated gene sets and hallmark gene sets: HUMMEL-BURKITTS_LYMPHOMA_UP, SHIPP_DLBCL_VS_FOLLICULAR_LYMPHOMA_UP, KEGG_LEUKOCYTE_TRANSENDOTHELIAL_MIGRATION, KEGG_CELL_ ADHESION_MOLECULES_CAMS, SCHOEN_NFKB_SIGNALING, GRAHAM_CML_ DIVIDING_VS_NORMAL_QUIESCENT_UP) were assessed for enrichment in our clusters. GSEA was run with the R package fgsea with a minimum pathway size of 15, a maximum pathway size of 500 and 100 permutations. A significance threshold of padj < 0.05 was applied to resultant pathway enrichments. scRNA-seq datasets were deposited at the ArrayExpress depository (ID:E-MTAB-10389).

#### Immunofluorescence staining of mouse tissue specimen

Organs were fixed overnight at 4°C in 4% PFA under permanent agitation. For immunofluorescence staining, LNs were embedded in 5% ultrapure low melting point agarose (Carl-Roth) in PBS and cut in 50-200 μm thick slices using a vibratome (VT-1200, Leica). LN sections from Ubow mice were treated using the CUBIC tissue clearing protocol (Nojima et al., 2017).

All sections were blocked and permeabilized in blocking buffer (10% normal goat or donkey serum, 0.1% Triton X-100 in PBS) for 1 hour at room temperature (RT). Primary antibodies were diluted in blocking buffer and incubated on tissue slides at 4°C ON. Secondary Alexa Fluor-conjugated antibodies were added and incubated for another 2 hours at RT. Microscopic recordings were performed using a LSM-710 laser scanning confocal microscope and a LSM-980 airyscan microscope with the ZEN blue edition software (all Carl Zeiss). Fiji image processing software and Imaris (v.9.7.2, Bitplane) were used for rendering, reconstruction, morphometric and volumetric analysis of image z-stacks and tile scans. No irregular nonlinear adjustments were performed and adjustments were only applied to whole images.

#### Immunohistochemistry of paraffin-embedded lymph nodes

For staining of murine PFA-fixed (ON at 4% PFA) LNs sections, tissues were dehydrated stepwise with 30%, 50%, 70%, 80%, 90%, and pure ethanol solution for 1 hour, respectively. After subsequent incubation in toluol, dehydrated LNs were embedded in paraffin and sectioned into 6 μm thick slices (HM355S Microtom, Microm).

Murine sections and human MTAs were heat treated at 60°C for 60 min. Prior to the staining procedure, paraffin was removed in xylol and acetone, and sections were rehydrated and treated with 10 mM citrate buffer for 5 min in a pressure cooker for antigen retrieval. All sections were blocked with avidin/biotin blocking solution (DAKO), incubated with the primary antibody overnight at 4°C and subsequently incubated with alkaline phosphatase (AP) or horse radish peroxidase (HRP) -conjugated secondary antibodies for 1 hour at RT. Fuchsin or 3-amino-9-ethylcarbazole (ACE) staining of sections was performed with the Fuchsin Substrate-Chromogen System or ACE substate (both DAKO) according to the manufacturer’s instructions. All sections were counterstained with Hematoxylin (ThermoFisher) and mounted with Kaiser’s glycerol gelatin (Merck Millipore). Bright field microscopy was performed using an Axio Imager 2 microscope and Axio Vision 4.8.2 Software (both Carl Zeiss).

#### Light sheet microscopy and whole organ analysis

Blood vessels were visualized using *Cdh5*^CreERT2^-*R26*^dTomato^ reporter mice. HEVs were detected by intravenous injection of Alexa-Fluor647 conjugated MECA-79 antibody (10 μg in 0.9% NaCl) 10 min prior to LN excision. Tissue clearing was performed according to the manufacturers guidelines (LifeCanvas technologies). In brief, mice were cardially perfused with PBS and SHIELD fixation solution (including 4% PFA, LifeCanvas technologies), followed by post-fixation in SHIELD fixation solution at 4°C overnight. Subsequent tissue processing and clearing was performed following the manufacturer’s instructions with Smart Clear Pro II (LifeCanvas technologies). Cleared LNs were imaged in Easy Index solution (refraction index 1.45, LifeCanvas technologies) using a Z.1 light sheet imager (Zeiss) with a 25x objective (immerged in Easy Index solution). Z-stacks were acquired in the multi-view tile scan mode with dual side illumination. Stitching, 3D reconstruction and resampling was performed using arivis Vision4D software (v.2.12). Visualization and quantification of the vessel network and HEVs was performed using the filament tracer module in Imaris (v.9.7.2, Bitplain).

#### Image analysis

##### FRC and conduit components

The fluorescence signal of *Ccl19*^RFP^ reporter mice was used to define the FRC network in LN vibratome sections. *Ccl19*^RFP^ network was segmented with the local background subtraction method in Imaris to remove background and compensate intensity differences between cell bodies and the branching extensions in the FRC network. The FRC network segmentation was used as template (masked) for the corresponding channels of the component of interest to analyze FRC associated mean fluorescence intensities (MFI). Collagen-I and CCL21 were analyzed in tile-stack scans of whole LN sections (50 μm in z-dimension). Collagen-IV and FITC-dextran were analyzed in regions (approximately 250x250 μm) at the border of the paracortex with minimal presence of blood vessels.

#### FITC-dextran in LN parenchyma

FITC-dextran in LNs was determined in tile-stack scans of whole LN sections (50 μm in z-dimension) as MFI of sum intensity projections using ImageJ (v2.1.0). FITC-dextran in the LN parenchyma was determined as MFI in regions (approximately 250x250 μm) at the border of the paracortex with minimal presence of blood vessels. Parenchyma was defined as volume outside of the FRC network (*Ccl19*^RFP^ segmentation and masking as described before) (Figure S7D).

#### Localization analysis of transmigrating lymphocytes

Lymphocytes from UBC^GFP^ donor mice were analyzed in recipient *Cdh5*^dTomato^ reporter mice to delineate blood vessels. Five random and GFP^+^ Lymphocyte-containing vessels were analyzed per mouse. Cell positions relative to the blood vessel were calculated as proportion of all GFP^+^ lymphocytes.

#### HEV associated expression analysis

HEVs segmentation (PNAd^+^) was used as template (mask channel option) to the corresponding channel to determine the MFI of the CCL21 staining in tile-stack scans of whole LN sections (50 μm in z-dimension). Autotaxin analysis was performed in *Cdh5*^dTomato^ reporter mice to identify blood vessels. Autotaxin expression was determined with line scans of the corresponding channel at the vessel lining.

#### Cdh5-Ubow reporter mouse analysis

YFP and CFP double positive voxels were combined to a new channel (YFP^+^CFP^+^) using the co-localization module in Imaris (v9.7 Bitplain). All channels were individually segmented and analyzed for the mean volumes of mono-colored clusters in capillary-like smaller vessels and HEV-like larger vessels.

#### HEVs in human tissue and spontaneous mouse lymphomas

HEVs were quantified by two independent investigators as count of PNAd^+^ vessels per biopsy core section in human MTAs or per whole LN section in mouse tumors (sp. Eμ-*Myc* and sp. *Cd19*xTAg).

#### Intravital 2-Photon microscopy

3x10^7^ freshly isolated lymphocytes from LNs and spleen of *UBC*-GFP mice were adoptively transferred (i.v.) into *Cdh5*^dTomato^ recipients. After 16 hours, the popliteal LN was exposed for two-photon-imaging as described before (Ulbricht et al., 2017). Mice and area of the exposed LNs were constantly monitored and heated to 37°C. Consecutive z-stacks were acquired over time periods of 30-50 min. Tracking of cells was performed using the spots module in Imaris (v.9.7.2, Bitplain).

#### Quantitative RT-PCR

Cells were lysed and homogenized using QIAGEN RLT buffer and Shredder columns before mRNA was extracted with the RNeasy Mini or Micro Kit (all QIAGEN). The cDNA was synthetized using the SuperScript VILO cDNA Synthesis kit (ThermoFisher), and gene expression was analyzed with the StepONE Plus PCR System by using pre-manufactured TaqMan primer together with the TaqMan Gene Expression Master Mix (all Applied Biosystems). The following TaqMan primer specificities were used (mouse *Ltb*: Mm00434774_g1, mouse *Actb*: Mm00607939_s1, human *LTB*: Hs00242739_m1, human *B2M*: Hs00187842_m1). Data were normalized to the housekeeping genes *Actb* or *B2M*.

#### Gene expression profiling and gene expression arrays

Microarray data are available at the Gene Expression Omnibus database under the accession numbers GSE126033 in Gloger et al. (2020), and GSE123593 in Scholz et al. (2020).

The DLBCL heterogeneity signature was generated based on microarray data from publically available datasets of DLBCL patients (Grau et al., 2019). Accession number of DLBCL microarray datasets GSE31312.

### Quantification and statistical analysis

Statistical data were evaluated using GraphPad Prism (v.6) software. The confidence level was 95%, with a significance level of 5% (α = 0.05). Results are expressed as the arithmetic means ± SEM. Data comparison with P values of ≤ 0.05 was considered statistically significant. P values were calculated by Wilcoxon signed-rank test, unpaired Students t test or Mann–Whitney U-test. Statistical details of the assays applied can be found in Figure legends.

## Data Availability

• Single-cell data have been deposited in the ArrayExpress data base: E-MTAB-10389 and are publicly accessible as of the date of publication. All original data reported in this paper is available from the Lead Contact upon request. • This paper does not report original code. • Any additional information required to reanalyze the data reported in this work is available from the Lead Contact upon request.

## Supplemental information

Document S1. Figures S1–S7

Document S2. Article plus supplemental information
