# Supplementary material for: Lymphocyte access to lymphoma is impaired by high endothelial venule regression
Source: Cell Rep. 2021 Oct 26;37(4):109878. doi: 10.1016/j.celrep.2021.109878 (PMC8567313; doi:10.1016/j.celrep.2021.109878)
Supplement: Document S1. Figures S1–S7 [file mmc1.pdf]

Cell Reports, Volume *37*

Supplemental information

Lymphocyte access to lymphoma is impaired by high endothelial venule regression

Lutz Menzel, Maria Zschummel, Tadhg Crowley, Vedran Franke, Michael Grau, Carolin Ulbricht, Anja Hauser, Volker Siffrin, Marc Bajénoff, Sophie E. Acton, Altuna Akalin, Georg Lenz, Gerald Willimsky, Uta E. Höpken, and Armin Rehm

Supplemental Figures

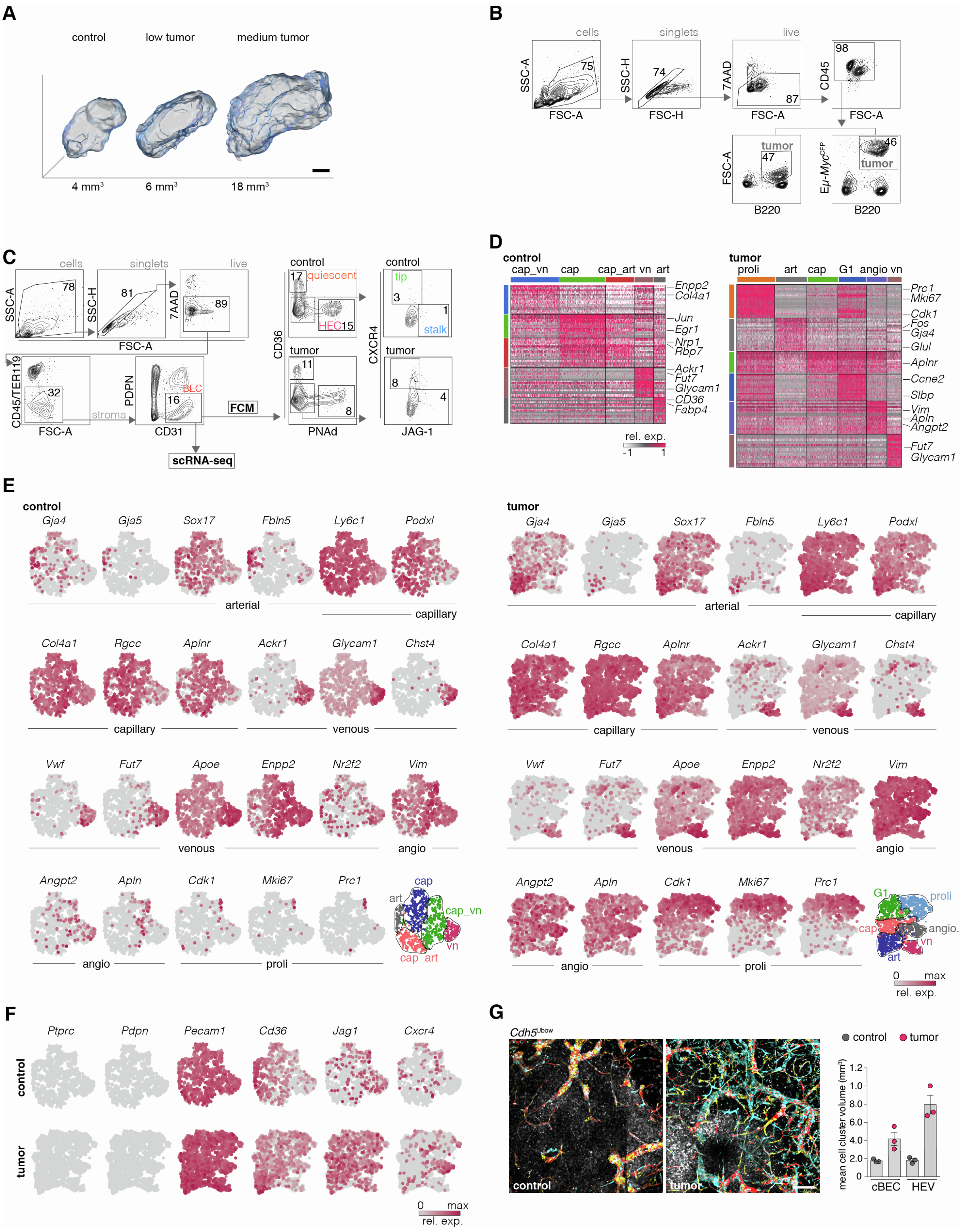

Figure S1, Lymphoma induced LN expansion and increased density of capillary-like blood vessels

Related to Figure 1. **A)** Representative image of inguinal LNs recorded by light sheet microscopy (LSM) (low 5-15 %; med 30-50 % tumor of CD45^{+} cells). Shown is 1 out of 3 individual experiments. **B)** Gating strategy of Eμ-*Myc* and Eμ-*Myc*^{CFP} tumor cells. **C)** Gating strategy of BECs for FCM analysis and sorting of BECs for scRNA-seq processing. **D)** Gene expression heatmaps of the top 20 diffentially expressed genes for each cluster.

E) t-SNE dimensional reduction depicts expression of subset associated markers. **F)** t-SNE dimensional reduction depicts marker expression used for FCM analysis at mRNA level in scRNA-seq data sets. **G)** *Cdh5*^{Ubow} mice were treated with tamoxifen and tumor was transferred 2 weeks later. 200 µm thick LNs sections were clarified, and imaged by confocal microscopy. Pictures show the appearance of contiguous and large monocolored segments of HEV cells only in tumor bearing LNs. Right, quantification of mono-colored cell clusters divided in classical BECs (cBEC) and HEVs, n=3 mice per group. Expression levels are indicated by the color scale (**D, E, F**). Mean and s.e.m. are indicated, data points represent individual animals (**G**). Scale bars, 500 µm (**A**), 50 µm (**G**).

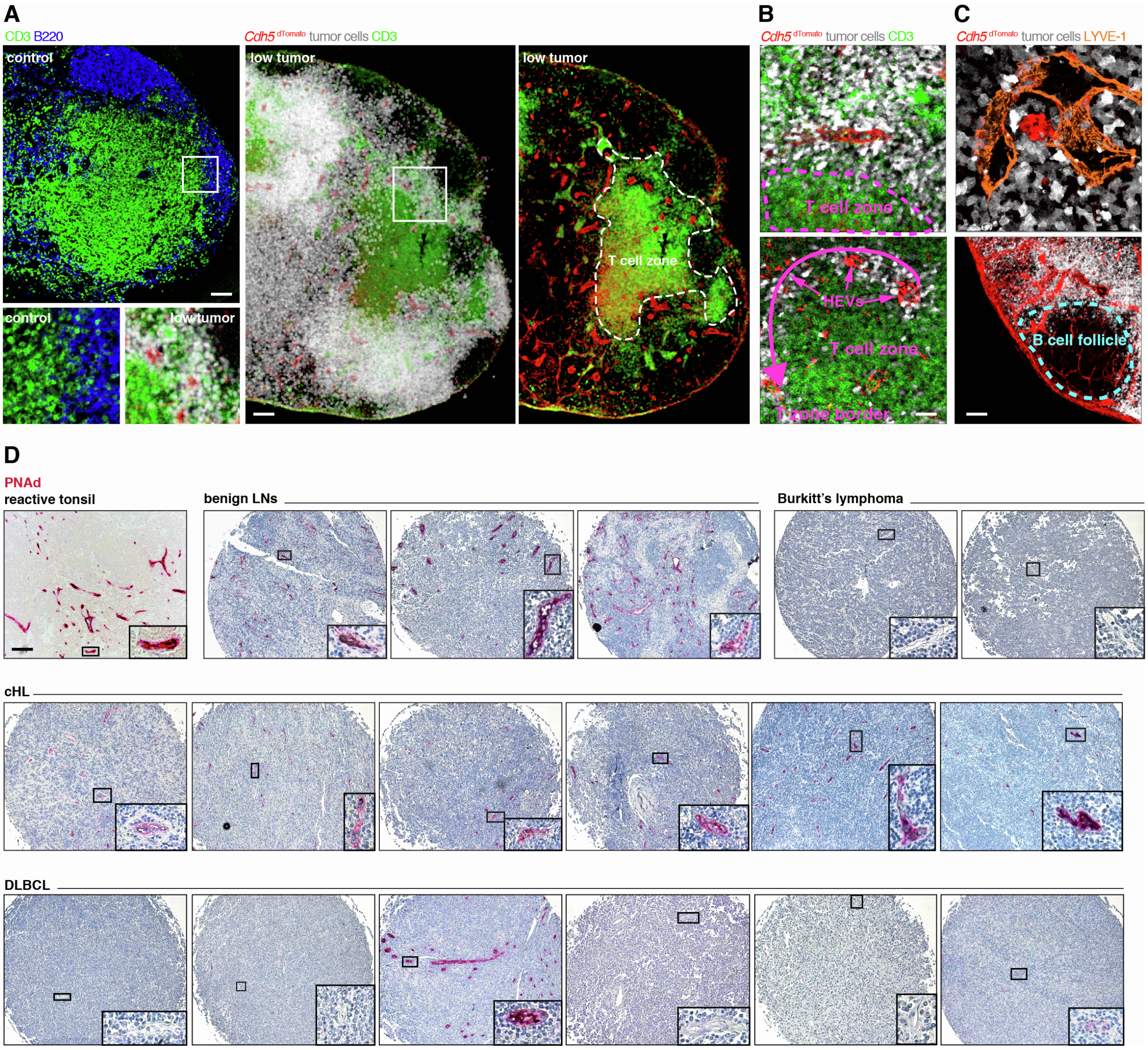

Figure S2, Lymphoma progression caused severe regression of HEVs in aggressive human B-NHLs

Related to Figure 2. **A)** Representative LN section from wt controls and *Cdh5*^{dTomato} mice with Eμ-*Myc*^{CFP} tumor cells and CD3 (T cells), B220 (B cells) staining. Right, channel of tumor cells not displayed to depict contracted T cell zone in LN with tumor. **B)** Representative LN sections from *Cdh5*^{dTomato} mice with Eμ-*Myc* tumor cells^{CFP} and CD3 staining, top, showing the separation of tumor and T cells at the border of the T cell zone, bottom, tumor cells surrounding HEVs. **C)** Representative LN sections from *Cdh5*^{dTomato} mice with Eμ-*Myc* tumor cells^{CFP} and LYVE-1 staining, top, showing the localization of tumor cells arounds HEVs and around lymphatic vessels, bottom, Eμ-*Myc* tumor cells are excluded from B cell follicles. **D)** Exemplary images of the immunohistochemical analysis of PNAd expression in a reactive tonsil, benign LNs, classical Hodkins lymphoma (cHL), diffuse large B cell lymphoma (DLBCL) and Burkitt's lymphoma (BL). Boxes indicate magnified areas (**A, D**). Scale bars, 100 μm (**A**, **D**), 50 μm (**D, C**).

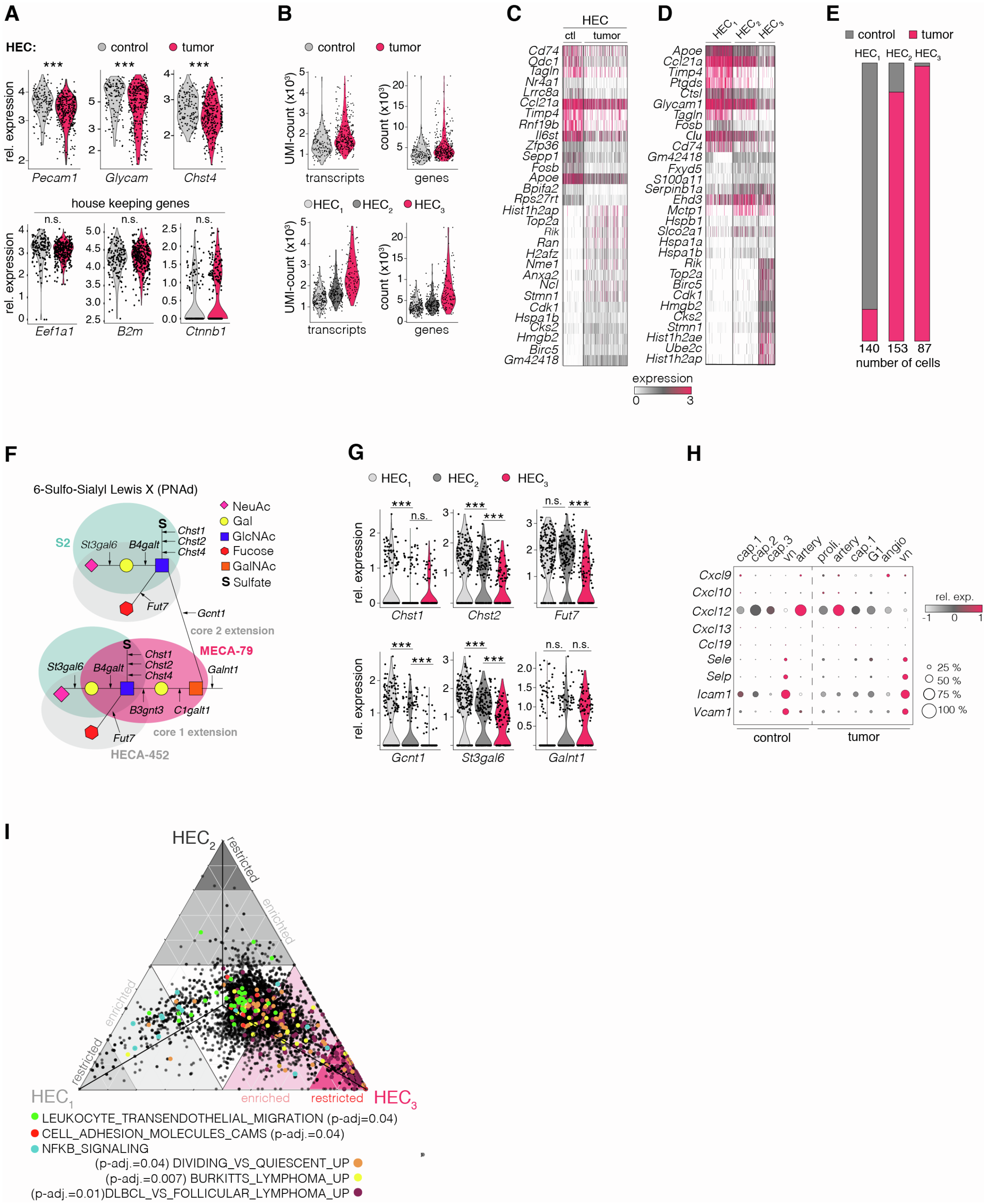

Figure S3, Lymphoma induces gradual dedifferentiation of HECs

Related to Figure 3. **A)** Violin plots depict expression of HEV markers and housekeeping genes in scRNA-seq data of HECs. HECs were selected from BECs by using HEC specific marker expression (*Glycam1* and *Chst4*). **B)** Violin plots depict count of detected transcripts (unique molecular identifiers, UMIs) and count of detected genes per group and clusters. **C)** Heatmap depicts most differentially expressed genes between HECs from control and tumor LNs and between the three HEC clusters. **D)** Heatmap depicts most differentially expressed genes between the HEC_{1-3} clusters. Expression changes are indicated by the color scale. **E)** Proportions of cells from control and tumor LNs within HEC_{1-3} clusters. **F)** Schematic illustration of the PNAd structure, including recognition sites of the PNAd binding antibodies MECA-79, HECA-452. **G)** Violin plots of genes coding for

enzymes that take part in the assembly of PNAd. **H)** Dot plot depicts the percentage of expressing cells and the expression level of genes that are associated with lymphocyte transendothelial migration in reactive LNs during inflammatory conditions. **I)** Ternary plot depicts relative association of genes to the different HEC clusters. Color attributes genes to selected enriched or negatively enriched gene sets. Statistics were calculated with Wilcoxon rank sum test (**A, G)**) (***p<0.005, n.s.=not significant).

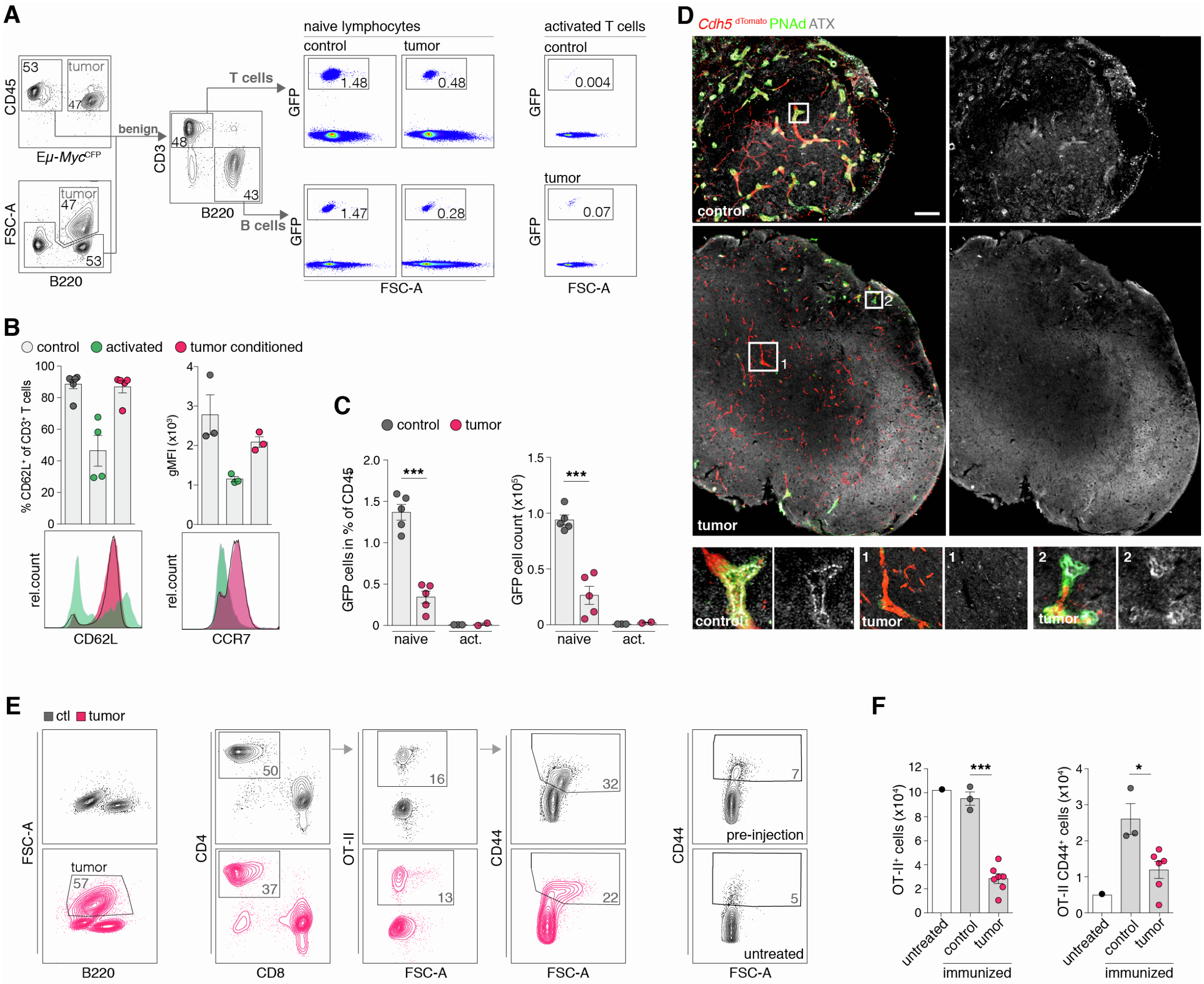

Figure S4, Lymphoma induce a non-permissive HEV condition for lymphocyte immigration.

Related to Figure 4. **A)** Gating strategy and representative plots of lymphocyte homing into the LN after adoptive transfer, analyzed by flow cytometry. **B)** LN homing receptor expression in non-activated versus CD3/CD28 activated T cells and T cells isolated from tumor bearing LN/spleen (tumor conditioned). **C)** LN homing of adoptively transferred non-activated lymphocytes and activated T cells. Data points represent individual animals (naïve n=5, activated n=2-3). **D)** Representative images of the autotaxin (ATX) expression in LNs. **E)** Flow cytometry analysis of OT-II cell infiltration and activation in draining LNs after OVA immunization. **F)** Cell count of OT-II^{+} T cells and OT-II^{+} CD44^{high} T cells in draining LNs after OVA immunization, n=3-7 mice per group. Boxes indicate magnified area. Scale bar, 100 µm. Mean and s.e.m. are indicated, data points represent individual mice (**B, C, F**). Statistics were calculated with Mann-Whitney U-test (***p≤0.005).

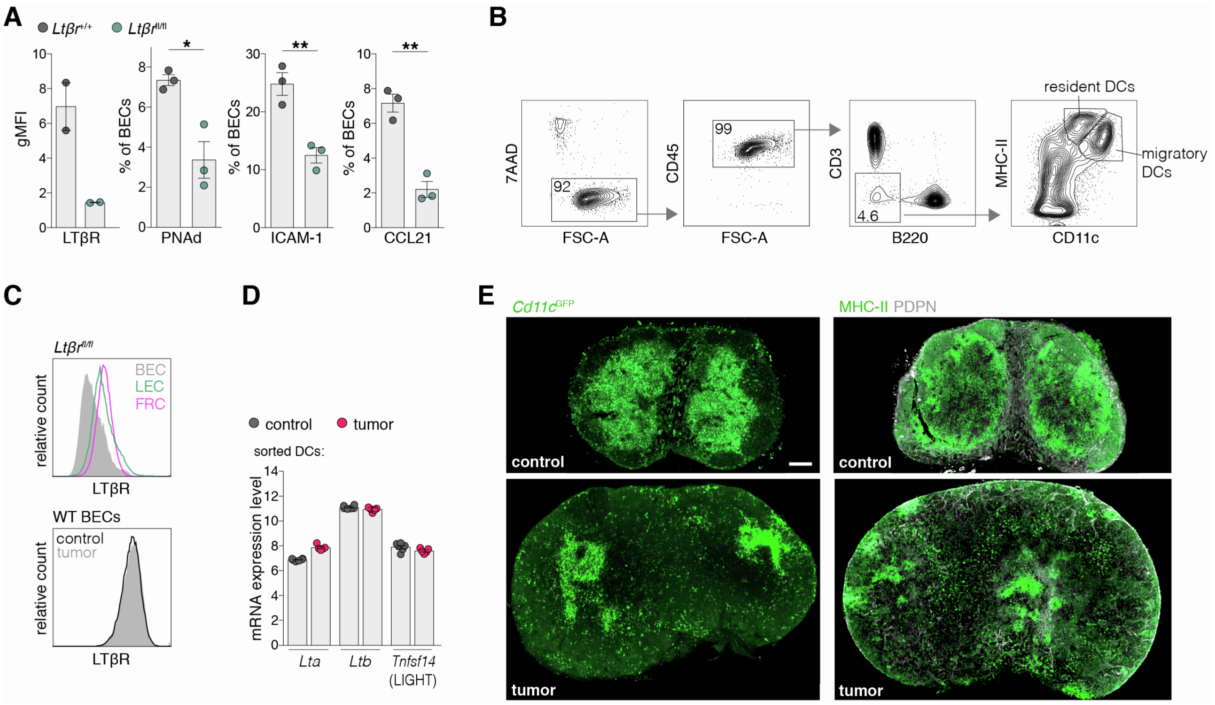

Figure S5, DCs provide HEVs with LTβR stimuli

Related to Figure 5. **A)** Flow cytometry analysis of BECs isolated from controls (*Ltbr*$^{+/+}$) or mice with conditional knock out of the *Ltbr* gene in endothelial cells (*Cdh5*^{CreERT2} x *Ltbr*$^{fl/fl}$, referred to as *Ltbr*$^{fl/fl}$) 10 days after first tamoxifen treatment. **B)** Gating strategy of the flow cytometry-based analysis of DCs. **C)** Top, representative histograms of the LTβR expression in stromal subsets in LNs of *Ltbr*$^{fl/fl}$ mice and, bottom**,** in BECs during control and tumor conditions. **D)** Gene expression data of DCs, isolated from SLOs of controls or mice with tumor. Measured by using gene expression microarrays. **E)** Representative images of the DC network in LN sections of *Cd11c*^{GFP} reporter mice or stained with MHC class II (MHC-II) antibody. Scale bar, 100 µm. Mean and s.e.m. are indicated. Data points represent individual animals (**A**) or experiments (**D**). Statistics were calculated with t-test (*p≤0.05, **p≤0.01).

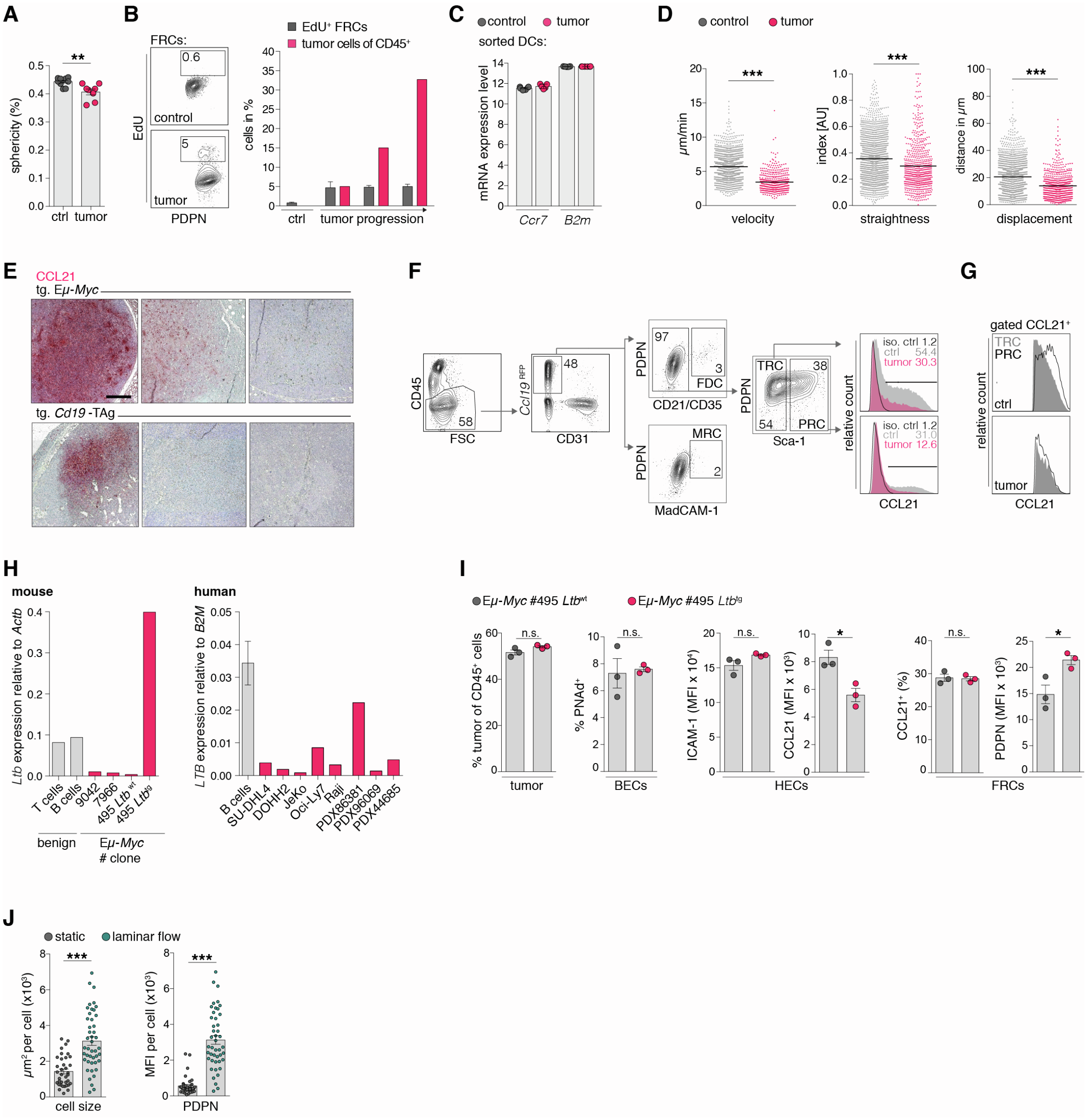

Figure S6, LN mesenchymal stroma cells require dual stimulation though LTβR signaling and mechanical input

Related to Figure 6. **A)** Quantification of the sphericity of FRCs, microscopically analyzed in LNs of *Ccl19*^{RFP} reporter mice, n=7 mice per group. **B)** Left, representative plots of *in vivo* EdU incorporation in FRCs analyzed by flow cytometry and, right, percent of EdU^{+} FRCs (7AAD^{-}CD45^{-}CD31^{-}PDPN^{+}) and percent of tumor cells (7AAD^{-}CD45^{+}FSC-A^{high}) relative to CD45^{+}, n=4 mice per group **C)** Gene expression data of DCs, isolated from SLOs and measured with gene expression arrays, n=3 per group. **D)** Lymphocyte migration behavior analyzed by intravital 2-photon imaging of popliteal LNs. Imaging was performed in the paracortex identified by the presence of HEV structures (HEV morphology in *Cdh5*^{dTomato} mice). The average track velocity, track straightness and the displacement from track origin were analyzed. Data points represent individual cells from 5 mice per group. **E)** Exemplary images of CCL21 expression in LN of transgenic (tg.) Eμ-*Myc* and *Cd19*-TAg mice. **F)** Flow cytometry gating strategy for FRC subsets and quantification of CCL21 expression. **G)** Histograms showing expression levels of CCL21^{+} gated FRCs. **H)** Gene expression analysis of benign

lymphocytes (n=4 donors), primary tumor cells, human NHL tumor cell lines and DLBCL patient derived xenograft (PDX) tumor cells. Measured by qRT-PCR. **I)** Quantification of the proportion of HECs (PNAd^{+} BECs), CCL21 expressing FRCs and PDPN expression levels (gMFI) in FRCs. Cells were isolated from mice with transferred Eμ-*Myc* tumor (#495-*Ltb*^{wt}) or tumor of the same clone transgenic for *Ltb* overexpression (#495-*Ltb*^{tg}). **J)** Immunhistological quantification of the cell size (area in μm^2) and PDPN expression (MFI) in a murine FRC cell line cultured under static conditions or with laminar flow. Mean and s.e.m. are indicated. Data points represent individual animals (**A, I**), experiments (**C**) or cells (**D**). Statistics calculated with Mann-Whitney U-test (**A**, **D, J**) or t-test (**I**) (*p≤0.05, **p≤0.01***p≤0.005; n.s., not significant).

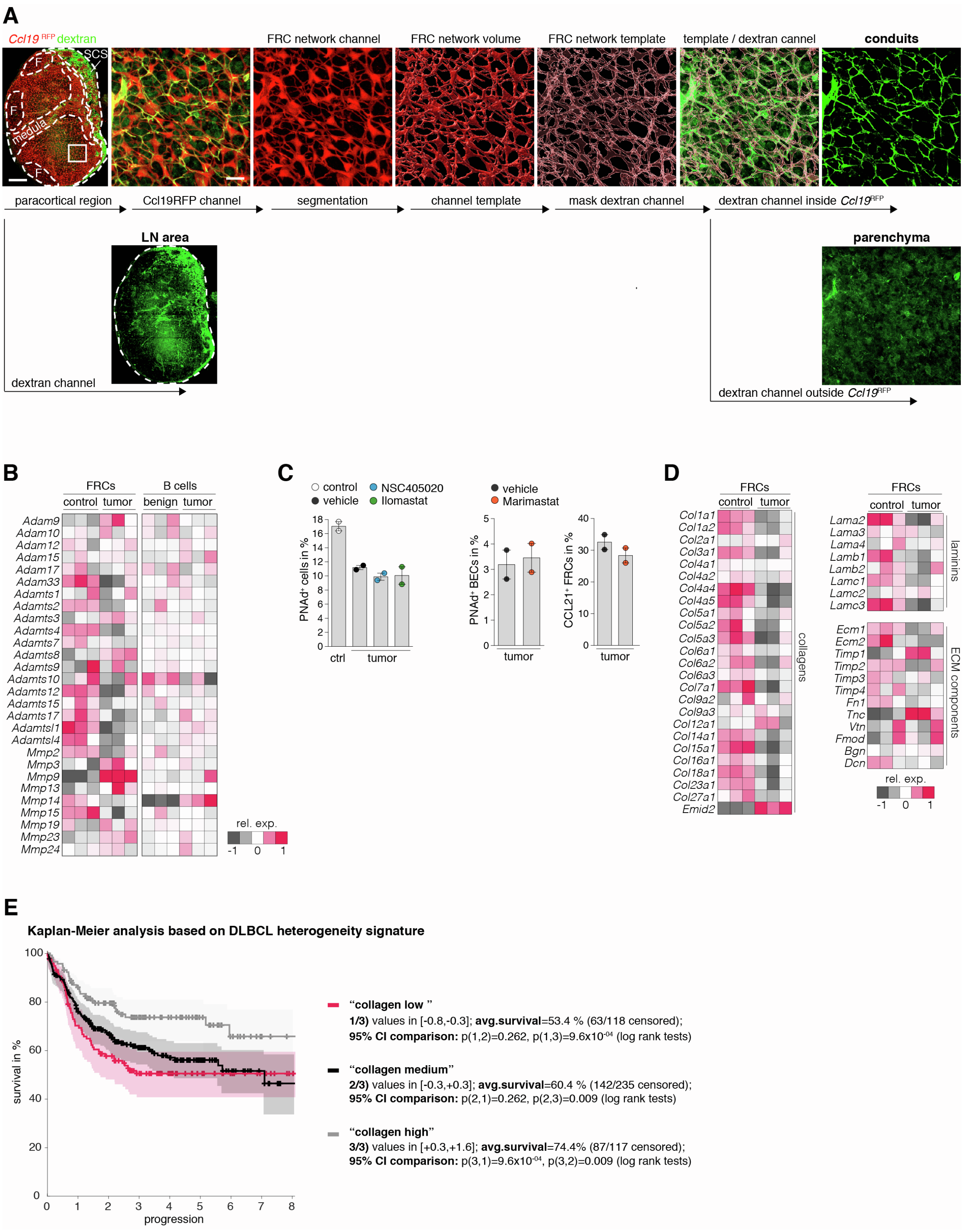

Figure S7, Lymphoma progression is accompanied by degeneration of the reticular conduit system

Related to Figure 7. **A)** Exemplary workflow of the image processing and quantification of subcutaneously administered dextran in draining LNs. **B)** Heatmap of genes encoding for MMPs; comparative expression analysis in FRCs, benign B cells and Eμ-*Myc* tumor cells. Measured by using gene expression microarrays. **C)** Flow cytometry analysis of the proportion of PNAd expressing BECs and CCL21 expressing FRCs isolated from mice with tumor and treated with different MMP inhibitors or vehicle, n=2 mice per group. Data points represent individual animals. **D)** Heatmaps showing differential expression of genes associated with conduit structures and

ECM components. Measured by using gene expression microarrays. **E)** Quartile Kaplan-Meier analysis of progression free survival (splits based on average signature expression in **Figure 7H**, merged center quartiles, 470/498 cases with follow-up data, R-CHOP therapy). Legends indicate average survival and signature expression ranges of the three partitions and significance of differences between partitions (log-rank tests). Mean and s.e.m. are indicated. Scale bars, 100 µm (**A,** left), 20 µm (**A,** right).
